# Biodegradable Magnesium-Based Implants in Sports Orthopedic Surgery: Advances in Alloy Design, Surface Engineering, and Translational Evidence

**DOI:** 10.3390/bioengineering13080926

**Published:** 2026-08-15

**Authors:** Georgi Raykov, Jakob Adolf, Benedikt Hochbein, Georgi Enev, Dimitar Tenev, Michail Dimitrov, Dimitar Raykov, Nikolay Dimitrov

**Affiliations:** 1 Klinik Sonnenhof, 3006 Bern, Switzerland; 2 University Specialised Hospital for Active Treatment in Orthopaedics “Prof. Boycho Boychev”, 1618 Sofia, Bulgaria; 3 Charité—Universitätsmedizin Berlin, 10117 Berlin, Germany; 4 University Hospital Zurich, 8091 Zurich, Switzerland; 5 Department of Orthopaedics and Traumatology, Faculty of Medicine, Medical University of Varna, 9002 Varna, Bulgaria

**Keywords:** magnesium, biodegradable implant, sports medicine, ACL reconstruction, interference screw, surface modification, osseointegration, additive manufacturing, imaging compatibility

## Abstract

Biodegradable magnesium (Mg)-based implants represent a paradigm shift in orthopedic biomaterials, offering temporary mechanical support, inherent osteogenic bioactivity, and elimination of hardware removal surgery. These properties are particularly attractive for sports orthopedic applications, including anterior cruciate ligament (ACL) reconstruction, rotator cuff repair, meniscal fixation, and osteochondral fragment refixation, where young, active patients demand rapid return to function and where permanent metallic hardware poses long-term risks of stress shielding, imaging artifact, and reoperation. Despite extensive preclinical evidence demonstrating that Mg-based interference screws promote fibrocartilaginous enthesis regeneration, attenuate peri-tunnel bone loss, and achieve biomechanical fixation comparable to titanium, no human clinical trial has yet evaluated Mg fixation devices for soft-tissue reconstruction in sports medicine. Meanwhile, clinical fracture fixation data from over 468 patients across multiple trials and a meta-analysis confirm complication rates equivalent to those of titanium. This narrative review synthesizes the current evidence on Mg alloy design, surface engineering strategies, preclinical sports medicine applications, clinical translation in fracture fixation, imaging compatibility, and the remaining barriers to clinical adoption in sports orthopedic surgery. By mapping the translational gap between promising animal data and the absence of clinical sports medicine trials, this review aims to guide future research priorities and accelerate the pathway toward clinical application of Mg-based devices in sports orthopedics.

## 1. Introduction

Orthopedic sports injuries are among the most common reasons for surgical intervention in young, physically active populations, and their incidence continues to evolve. A national analysis of U.S. emergency department data demonstrated notable trends in sports-related upper extremity injuries over the past decade [1], with hand and wrist injuries in particular showing rising rates across age and sex subgroups from 2020 to 2024 [2]. These injuries, which include ligament ruptures, meniscal tears, osteochondral defects, tendon avulsions, and acromioclavicular joint dislocations requiring coracoclavicular ligament reconstruction [3], generate substantial demand for reliable fixation devices. The fixation devices used in these procedures (interference screws, suture anchors, pins, and staples) must provide reliable mechanical support during biological healing while minimizing long-term complications [4]. Current fixation options include permanent metallic implants (titanium, stainless steel) and biodegradable polymers (poly-L-lactic acid [PLLA], polyglycolic acid [PGA], and their copolymers), each with well-documented limitations.

Permanent metallic implants provide excellent initial fixation but cause stress shielding due to elastic modulus mismatch (titanium ~110 GPa vs. cortical bone ~15–30 GPa), generate significant MRI artifacts that impair post-operative imaging surveillance, and frequently require hardware removal, a second surgery that carries its own morbidity and cost [5,6]. In a first-in-humans study of bioresorbable Mg-Zn-Ca screws for medial malleolar fractures, 71% of patients who also received titanium fixation for associated fracture components required a second surgery for titanium hardware removal, while none of the Mg screws required removal [7]. Biodegradable polymer screws avoid reoperation for hardware removal but degrade into acidic by-products that can provoke sterile osteolysis, foreign-body reactions, and cyst formation; a 13-year randomized controlled trial (RCT) of PLLA-hydroxyapatite interference screws for ACL reconstruction demonstrated complete screw resorption but incomplete ossification of tibial tunnels [8].

The principal material classes currently available for orthopedic fixation, together with the biodegradable Mg alloys that are the subject of this review, are compared in Table 1, which summarizes their mechanical behavior relative to bone, degradation status, imaging characteristics, and clinical maturity.

Placed side by side, the five material classes each answer only part of the requirement set for sports orthopedic fixation, and each carries a defining weakness. Permanent metals deliver the most reliable initial fixation and the longest clinical track record, but their high elastic modulus (titanium ~110 GPa against cortical bone ~15 to 30 GPa) drives stress shielding, they generate the largest imaging artifact, and they are bioinert, so that osseointegration proceeds without any positive biological signal and removal is frequently undertaken as a second procedure with its own morbidity and cost [5,6,7,9]. Biodegradable polymers remove the removal operation but substitute a different problem: hydrolysis into acidic by-products that can provoke sterile osteolysis, foreign-body reaction, and cyst formation, together with slow or incomplete resorption and, in long-term ACL data, incomplete tunnel ossification [8]. Bioactive ceramics and glasses are the most biologically active of the non-metals, being osteoconductive and osteostimulative through their ionic dissolution products, but their brittleness and low fracture toughness confine them to non-load-bearing graft-substitute roles rather than primary fixation [15,16]. Carbon-fiber-reinforced PEEK narrows the mechanical gap, with a modulus (~16 to 24 GPa) close to bone and radiolucency that preserves imaging, yet it remains permanent and bioinert, offering neither resorption nor osteogenic stimulation [17,18]. Against this background, magnesium alloys are distinctive less for excelling on any single axis than for combining several: a bone-matched modulus (10 to 30 GPa) that limits stress shielding, in vivo resorption that in principle obviates removal, intrinsic osteoinductive signaling through released Mg^2+^ (Section 1 and Section 4), and lower MRI artifact that decreases further as the implant degrades [5,6,10,11,12,13,14]. That combination is what no other single class currently provides. It is tempered, however, by magnesium’s own principal weakness, namely rapid and non-uniform early corrosion with hydrogen-gas evolution and consequent loss of mechanical strength at load-bearing sites, which is the central barrier addressed in Section 7 and the reason clinical maturity remains confined to fracture fixation rather than the soft-tissue-to-bone sports indications that motivate this review.

Biodegradable magnesium alloys have emerged as a “third-generation” biomaterial that addresses many of these limitations. Mg possesses an elastic modulus (10–30 GPa) closely matching cortical bone, thereby minimizing stress shielding [5,6]. It degrades in vivo into non-toxic Mg^2+^ ions, the fourth most abundant cation in the human body, which are actively involved in bone metabolism, enzymatic reactions, and cellular signaling [10]. Critically, Mg^2+^ ions have been shown to stimulate osteogenesis through upregulation of BMP-2, VEGF, and activation of the canonical Wnt/β-catenin signaling pathway, making Mg implants not merely bioinert but actively osteoinductive [11,12]. Furthermore, Mg implants produce significantly fewer MRI artifacts than titanium, with artifact production decreasing further as the implant degrades, a property of particular importance in sports medicine, where serial MRI is essential for monitoring graft maturation and detecting complications [13].

The first biodegradable Mg orthopedic implant (MAGNEZIX^®^ compression screw, Syntellix AG, Hannover, Germany) received CE mark approval and entered clinical use in 2013 [19]. Since then, clinical data from over 468 patients across multiple trials and a meta-analysis have demonstrated complication rates equivalent to titanium for fracture fixation applications [20]. However, the translation of Mg-based devices into sports orthopedic surgery, particularly for soft-tissue-to-bone fixation in ACL reconstruction, rotator cuff repair, and meniscal surgery, remains at the preclinical stage.

This narrative review provides a comprehensive synthesis of the current evidence on Mg-based implants relevant to sports orthopedic surgery, organized into seven thematic sections: alloy design and development (Section 2), surface engineering strategies (Section 3), preclinical evidence in sports medicine applications (Section 4), clinical translation in fracture fixation (Section 5), imaging compatibility (Section 6), challenges, limitations, and future directions (Section 7), and the translational gap between preclinical promise and clinical evidence (Section 8), outlining priorities for future research.

Several reviews of Mg-based orthopedic biomaterials already exist, and it is therefore reasonable to ask what a further synthesis adds. Earlier reviews are organized predominantly around alloy metallurgy, corrosion behavior, and general fracture-fixation performance: general Mg-biomaterial and orthopedic-Mg overviews concentrate on modulus matching, degradation control, and load-bearing fixation [5,6,21,22], and the road-to-clinic overview frames the field chiefly in terms of regulatory translation for fracture indications [23]. The present review is instead organized around sports-orthopedic, soft-tissue-to-bone applications (ACL reconstruction, rotator cuff repair, meniscal fixation, and osteochondral refixation), where the biological demand is enthesis regeneration rather than diaphyseal or metaphyseal bone union. Within that frame, it synthesizes the molecular mechanisms of Mg^2+^ at the tendon–bone interface (Section 4), integrates imaging compatibility as a decision-relevant property for a field that depends on serial MRI (Section 6), and explicitly maps the translational gap between the preclinical sports-medicine evidence base and the absence of any human sports-medicine trial (Section 8). This soft-tissue-oriented, mechanism-to-imaging-to-trial-gap organization is the intended contribution; it is complementary to, rather than a repetition of, the metallurgy- and fracture-centered reviews cited above.

**Figure 1:**
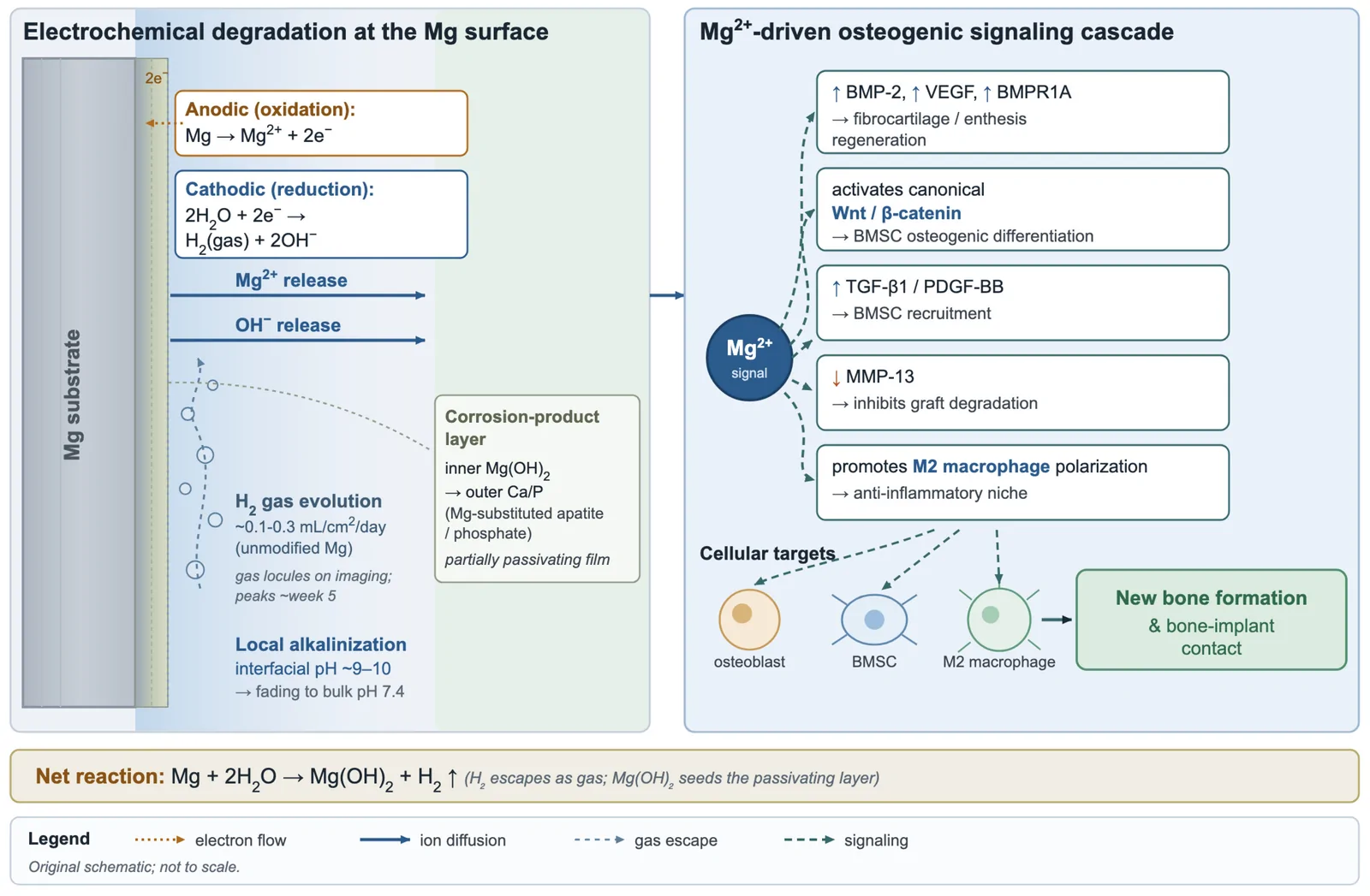
Electrochemical degradation of a magnesium-based implant and its downstream osteogenic signaling. Anodic dissolution (Mg to Mg^2+^ plus 2e^−^) is balanced by cathodic water reduction (2H_2_O plus 2e^−^ to H_2_ plus 2OH^−^), giving the net reaction Mg plus 2H_2_O to Mg(OH)_2_ plus H_2_. Hydrogen gas evolves (about 0.1 to 0.3 mL/cm2 per day for unmodified magnesium, producing gas locules on imaging that peak near week 5); the interfacial pH rises to about 9 to 10, and a corrosion-product layer (inner Mg(OH)_2_ grading to an outer calcium phosphate phase) forms at the surface. Released Mg^2+^ drives an osteogenic signaling cascade (upregulation of BMP-2, VEGF, BMPR1A, TGF-beta1 and PDGF-BB, activation of canonical Wnt/beta-catenin, downregulation of MMP-13, and M2 macrophage polarization) acting on osteoblasts, bone marrow stromal cells and macrophages to promote new bone formation and bone–implant contact. Arrows denote electron flow, ion diffusion, gas escape, and molecular signaling, as distinguished in the in-figure legend. Original schematic, not to scale.

Figure 1 summarizes the electrochemical degradation of a magnesium implant and the osteogenic signaling it drives.

## 2. Magnesium Alloy Design and Development

The selection of an appropriate Mg alloy system is fundamental to implant performance, as alloying elements determine mechanical strength, corrosion rate, biocompatibility, and biological activity. Five major alloy families have been investigated for orthopedic applications, each with distinct advantages and limitations [24].

**Figure 2:**
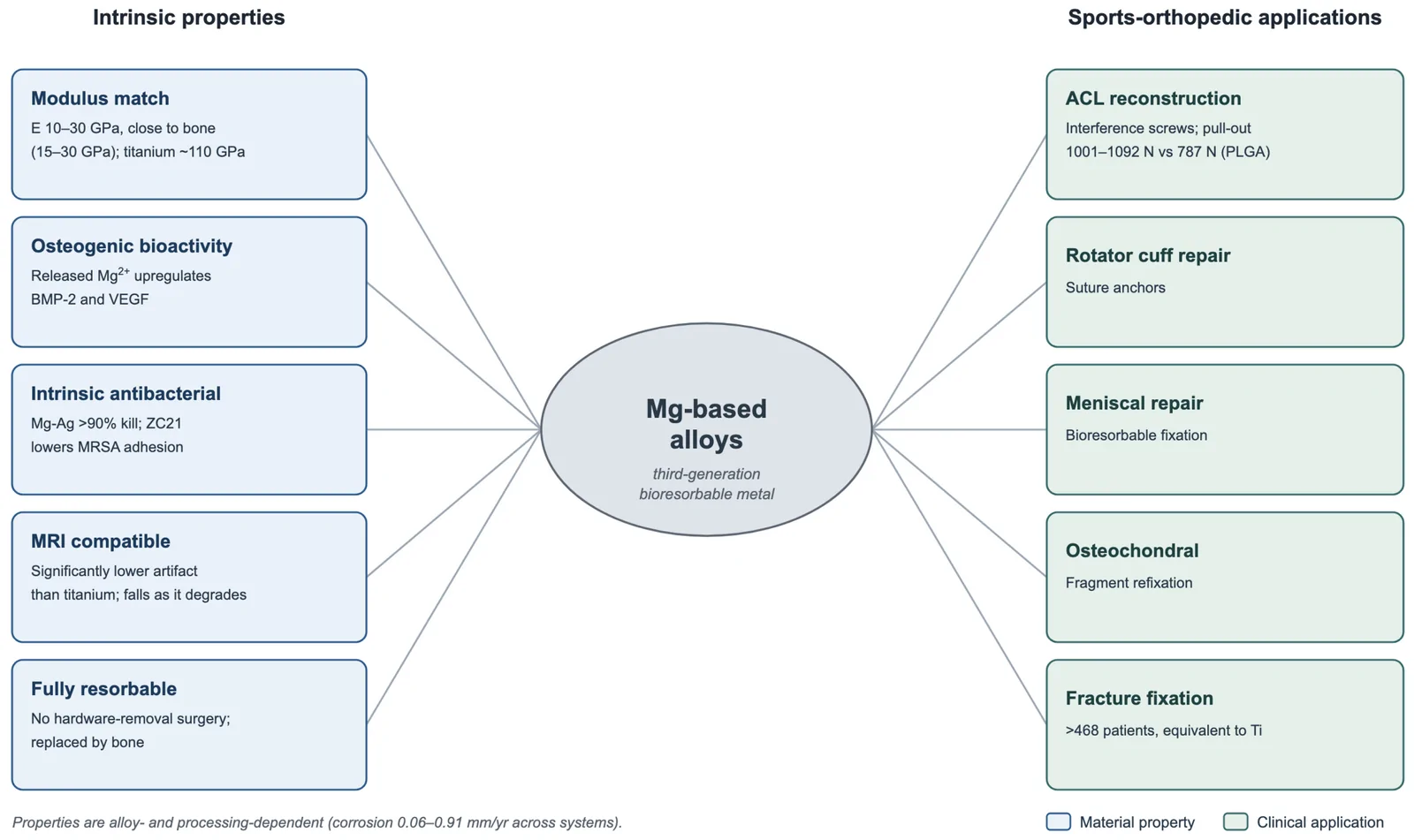
Hub-and-spoke overview of magnesium alloy properties and their sports-orthopedic applications. Five intrinsic properties (a bone-matched elastic modulus of 10 to 30 GPa versus about 110 GPa for titanium, Mg^2+^-driven osteogenic bioactivity, intrinsic antibacterial activity in selected systems, low MRI artifact, and full resorbability) map onto the principal sports indications: ACL reconstruction, rotator cuff repair, meniscal repair, osteochondral fragment refixation, and fracture fixation. Properties are alloy- and processing-dependent (corrosion rate 0.06 to 0.91 mm/yr across systems).

The intrinsic properties of magnesium alloys and their mapping onto specific sports-orthopedic applications are summarized in Figure 2.

### 2.1. Mg-Zn-Ca System

The Mg-Zn-Ca system uses only elements naturally present in the human body, eliminating concerns about rare-earth element toxicity [21,25]. Representative compositions range from ultra-lean alloys (ZX00: Mg-0.45Zn-0.45Ca wt.%) to moderately alloyed variants (ZC21: Mg-2Zn-0.5Ca wt.%) [25,26]. Mechanical properties are highly processing-dependent: hot-rolled ZX10 achieves a yield strength (YS) of ~121 MPa and an ultimate tensile strength (UTS) of ~226 MPa, while extruded Mg-1.2Zn-0.32Ca-0.25Sn (ZXT100) reaches YS ~345 MPa through grain-boundary strengthening [27,28]. Corrosion rates are among the lowest of any Mg alloy system, ranging from 0.06 mm/yr (hot-rolled ZX10) to 0.21 mm/yr (ZXT100) [27,28].

The ZX00 alloy has advanced furthest toward clinical translation within this family. In a sheep diaphysis model, ZX00 screws demonstrated controlled degradation over 24 weeks with new bone formation in direct contact with corrosion products and no adverse tissue effects [25]. A first-in-humans study of ZX00 screws for medial malleolar fractures (*n* = 20) reported all patients pain-free at 12 months, with no screws requiring surgical removal [7]. At the 2.5-year follow-up, homogeneous bone texture was observed in 15/18 patients evaluated (see Section 5.2 for full clinical and radiographic outcomes) [29].

The ZC21 alloy has demonstrated an additional advantage relevant to sports medicine: reduced MRSA bacterial adhesion compared to pure Mg, along with higher bone marrow stromal cell (BMSC) adhesion density and promoted osteogenesis in a mouse femoral defect model [26].

### 2.2. Mg-Y-RE-Zr System (WE43)

WE43 (Mg-4Y-3RE-0.5Zr, where RE = Nd, Ce, La mischmetal) is the most clinically advanced Mg alloy, forming the basis of the CE-marked MAGNEZIX^®^ compression screw [22,30]. Rare-earth elements improve creep resistance, mechanical strength, and corrosion resistance through solid-solution and precipitation strengthening [31,32]. Warm-rolled WE43 achieves UTS ~347 MPa with elongation ~8.9% and a corrosion rate of ~0.16 mm/yr, while ultrafine-grained WE43 produced by multiaxial deformation reaches UTS ~300 MPa with elongation ~17.2%, and its fatigue strength increases from 90 to 165 MPa [33,34].

PEO surface modification of WE43 screws in miniature pigs demonstrated a reduction of ~50% in corrosion rate at 6 months (residual screw volume 62.9% vs. 28.8% for unmodified screws, *p* = 0.027) and a nearly 3-fold increase in bone–implant contact area (51.6% vs. 18.1%, *p* = 0.015) [35]. Both as-cast and T5 heat-treated WE43 showed excellent biocompatibility and osteogenesis-promoting properties in sheep cranial models, with no Mg accumulation in lymph nodes [36].

However, concerns persist regarding the biological safety of high rare-earth content (~7 wt.%). Single-phase Mg-RE alloys with <1 wt.% Ho, Er, or Lu showed comparable in vitro and in vivo performance to high-purity Mg without local or systemic toxicity, with RE elements gradually excreted via the urinary and digestive systems [32]. A recent study in hyperlipidemic (ApoE^−^/^−^) mice found dramatically accelerated WE43 wire corrosion (wire breakage in 50% of implants at 10 days vs. 0% in wild-type), raising important questions about alloy performance in patients with metabolic comorbidities [37].

### 2.3. High-Purity Magnesium (HP-Mg)

HP-Mg (≥99.9–99.99 wt.%) eliminates concerns about alloying element toxicity and reduces micro-galvanic corrosion by removing second phases and impurities, but this comes at the cost of lower mechanical strength [38,39]. HP-Mg extracts promoted cell viability, bone ALP activity, and mRNA expression of osteogenic genes (ALP, OPN, RUNX2) in human BMSCs [39]. In vivo, 99.99% HP-Mg screws showed uniform degradation with increased new bone formation and bone–implant contact at 26 and 52 weeks, with no abnormal serum/urine Mg levels or organ dysfunction [38]. HP-Mg screws have been clinically used in China for osteonecrosis of femoral head and femoral neck fractures [22,40].

### 2.4. Mg-Li System

Lithium transforms the Mg crystal structure from HCP to BCC at >10.3 wt.% Li, dramatically improving ductility, addressing a critical limitation for complex implant geometries [41,42]. Extruded Mg-Li-Zn ternary alloys (LZ61, LZ91) achieve >40% elongation at fracture without significantly compromising strength [43]. Mg-14Li forms a single-phase β-Li structure with homogeneous, slow corrosion and a compact protective Li_2_CO_3_/Ca(OH)_2_ film, avoiding the severe pitting seen in conventional alloys [44]. However, lithium’s narrow therapeutic window (0.6–1.2 mEq/L serum) raises safety concerns for orthopedic applications, where larger implant volumes may release more Li than cardiovascular stents [41]. To date, Mg-Li alloys have been studied primarily for cardiovascular stent applications rather than orthopedic fixation.

### 2.5. Mg-Ag System

Silver possesses well-established antibacterial properties, addressing the clinical challenge of implant-associated infections. Mg-Ag alloys achieve killing rates exceeding 90% against *S. aureus* and *S. epidermidis* in dynamic bioreactor assays with negligible cytotoxicity to primary osteoblasts [45]. The Mg-Ag-Y alloy demonstrates a degradation rate of ~0.91 mm/yr in vivo (vs. 1.80 mm/yr for pure Mg) with significantly more new bone formation (3.18 mm^3^ vs. 1.39 mm^3^ at 6 weeks in rat femur) [46]. These alloys remain at the preclinical stage but offer a promising approach for infection-prone sports medicine applications.

### 2.6. Comparative Overview of Alloy Systems

Table 2 summarizes the representative compositions, mechanical properties, corrosion rates, and clinical status of the five alloy systems.

A critical cross-cutting observation is that thermomechanical processing (extrusion, rolling, ECAP, heat treatment) can shift yield strength by 2–3x and corrosion rate by an order of magnitude within any alloy system, making direct cross-system comparisons challenging without specifying the processing state [28,33,34].

### 2.7. Mechanical Performance Requirements for Sports Orthopedic Fixation

The mechanical demands placed on a fixation device in sports orthopedic surgery differ substantially from those in load-bearing diaphyseal fracture fixation, and they differ among sports procedures themselves. An interference screw for ACL reconstruction is loaded primarily in a shear/friction mode at the graft-tunnel interface and must resist graft pull-out under both a single supraphysiological traction and thousands of submaximal cycles as the patient rehabilitates, whereas a suture anchor is loaded in tension along the suture axis, and meniscal or osteochondral fixation devices experience comparatively low static and compressive loads while cartilage or meniscal tissue heals. None of these constructs is expected to carry the full weight-bearing bending and torsional loads that dominate long-bone fracture fixation, so the governing requirement is retention of adequate interface strength during a defined soft-tissue-to-bone healing interval rather than sustained load transfer across a fracture gap. This distinction matters for Mg because the alloy families summarized in Section 2.6 span a wide mechanical range, and the appropriate target strength depends on which of these load environments the device must serve.

For soft-tissue-to-bone fixation, the available preclinical benchmarks are encouraging. Ex vivo testing of MgYREZr-alloy interference screws produced maximum pull-out forces of 1001 to 1092 N, significantly higher than commercial PLGA Milagro screws at 787 N (*p* < 0.05), and cyclic loading over 1000 cycles at 0 to 200 N showed no significant difference in pull-out force between foam-block and cadaveric setups, indicating that initial fixation is preserved under repeated submaximal loading [47,48] (see Section 4.1). Time-dependent behavior is equally relevant: Mg-6Zn-0.5Sr interference screws that degraded completely within 12 weeks in a rabbit ACL model nonetheless showed that maximum load to failure significantly improved at 16 weeks, as bony ingrowth replaced the resorbing screw and attenuated peri-tunnel bone loss relative to PLA controls [49]. In a non-fixation push-out configuration, a WE43-based alloy achieved significantly greater maximum push-out force, ultimate shear strength, and energy absorption than titanium at 4, 12, and 24 weeks, alongside higher bone–implant contact [50] (Section 7.2), which suggests that the biological contribution of Mg to interface strength can offset the loss of implant substance during degradation.

The central tension is that a Mg device must retain sufficient mechanical integrity across the roughly 6- to 12-month soft-tissue-to-bone healing window while it is simultaneously corroding, and that the mode of corrosion, not only its average rate, determines whether that requirement is met. Early resorption within the first 3 months combined with inadequate residual strength is the principal obstacle to load-bearing use, and non-uniform pitting corrosion is especially problematic because it produces abrupt, localized deterioration in strength rather than a gradual, predictable decline [51,52] (Section 7.1). Alloying and thermomechanical processing provide the main levers to meet a given target: within the compositions of Section 2, yield strength ranges from approximately 121 MPa for hot-rolled ZX10 to approximately 345 MPa for extruded ZXT100, so a procedure demanding high initial fixation can in principle be matched to a higher-strength, more corrosion-resistant alloy and processing state, whereas a lower-load meniscal or osteochondral application tolerates a leaner, faster-resorbing composition. Where the required initial fixation exceeds what a resorbing Mg construct can reliably sustain, particularly at more load-exposed sites, Mg/Ti hybrid fixation systems that pair a permanent load-bearing element with a bioactive degradable Mg component, or the use of higher-strength alloys, offer a means of decoupling immediate mechanical stability from the osteogenic and degradation behavior of Mg [53] (Section 7.6).

## 3. Surface Engineering Strategies

Unmodified Mg alloys corrode at rates exceeding 2 mm/yr, with hydrogen gas evolution of 0.1–0.3 mL/cm^2^/day in physiological environments, which is far too rapid for clinical bone healing timelines [5]. Surface engineering is the primary strategy to control degradation while enhancing bioactivity [54].

### 3.1. Micro-Arc Oxidation/Plasma Electrolytic Oxidation (MAO/PEO)

MAO generates a ceramic oxide layer (primarily MgO, Mg_3_(PO_4_)_2_, and/or MgSiO_3_) directly on the Mg surface through plasma discharge at high voltage. A systematic review found that MAO reduces corrosion rates by approximately 60–85%, from >2 mm/yr to 0.3–0.8 mm/yr [5]. Frequency-controlled AC-MAO processing on pure Mg achieves a corrosion rate of 0.012 mm/yr (from 5.50 mm/yr for bare Mg) [55].

The most robust in vivo evidence comes from an 18-month Göttingen miniature pig study of ZX00 screws with PEO surface modification, which demonstrated significantly slower and more uniform degradation alongside enhanced bone formation compared to non-modified screws [56]. The incorporation of bioactive elements (cerium oxide nanoparticles, zinc, gallium) into MAO layers further enhances osteogenic and tribological properties [57,58]. The inherent porosity of MAO coatings, however, necessitates post-treatment sealing strategies for optimal corrosion protection [59,60].

### 3.2. Hydroxyapatite and Calcium Phosphate Coatings

HA coatings achieve corrosion rates of approximately 0.25 mm/yr while providing osteoconductivity through chemical similarity to bone mineral [5]. A comparative study of Mg(OH)_2_, MgF_2_, and HA coatings found that HA exhibited the best in vitro corrosion resistance and the best in vivo osseointegration, with the lightest inflammatory response in rat femoral implantation [61]. HA-coated Mg screws showed significantly retarded biocorrosion in vivo, particularly over the first 6 weeks, which considerably promoted bone growth at the bone–implant interface [62]. Fluoridated hydroxyapatite (FHA) coatings on Mg-Zn-Ca alloys showed enhanced stability and improved early bone mineralization [63].

### 3.3. Fluoride Conversion Coatings

Fluoride conversion treatment produces a thin MgF_2_ layer (1.5–5.5 μm) that provides effective initial corrosion protection. MgF_2_-coated screws showed corrosion rates decreasing from 0.49 mm/yr initially to 0.01 mm/yr after 24 h [64]. In vivo, fluoride-coated AZ31B screws upregulated BMP-2 and collagen type I expression, confirming osteogenic activity [65]. However, the MgF_2_ layer provides primarily short-term protection (~4 weeks in vivo), making it most useful as a priming layer for duplex coating systems [66]. MgF_2_/PCL duplex coatings showed improved corrosion resistance and cell viability, and reduced in vivo degradation compared to single MgF_2_ or uncoated alloy [67].

### 3.4. Biodegradable Polymer Coatings

Polymer coatings (PLA, PLGA, PCL, polydopamine, chitosan) provide a physical barrier while being biodegradable themselves [68]. PCL-coated Mg showed reduced degradation, maintained mechanical properties, and higher volumes of new bone without inflammation or hydrogen gas accumulation in vivo [69]. A systematic comparison found that PLGA (50:50) most improved endothelial cell adhesion and spreading, attributed to favorable Mg ion release (9–15 mM range) [70]. Polydopamine serves as a versatile intermediate adhesion layer for further functionalization, enabling immobilization of HA nanoparticles and BMP-2 with enhanced corrosion resistance and new bone formation in vivo [71]. pH-responsive chitosan-based smart coatings achieved 97.27% corrosion inhibition efficiency while promoting osteoblast proliferation and differentiation [72].

### 3.5. Composite and Multilayer Systems

The most promising strategies combine multiple coating layers. MAO/PLLA composites on Mg-1Li-1Ca significantly enhanced corrosion resistance, with PLLA degradation products (lactic acid) buffering the pH increase from Mg corrosion [73]. PEO-CaP duplex coatings showed corrosion current density ~99% lower than bare Mg [74]. A sandwiched Mg(OH)_2_/stearic acid/poly(trimethylene carbonate) coating achieved a 1.7-fold improvement in newly formed bone volume after 12-week implantation [75]. Self-healing polymeric coatings incorporating simvastatin-loaded mesoporous silica nanoparticles demonstrated water-enabled self-healing capacity with pH/NIR-responsive antibacterial activity and enhanced osteogenesis [76].

### 3.6. Summary of Coating Performance

Table 3 summarizes the corrosion-rate reduction, principal biological effects, and characteristic limitations of the main coating strategies.

## 4. Preclinical Evidence in Sports Medicine Applications

### 4.1. ACL Reconstruction: Interference Screws

The largest body of preclinical evidence for Mg in sports medicine comes from ACL reconstruction models. Two systematic reviews confirm that Mg-based interference screws are among the most promising osteoinductive fixation strategies for enhancing tendon graft–bone healing [77,78].

**Enthesis regeneration and molecular signaling**. Cheng et al. (2016) [11] demonstrated that HP-Mg interference screws fixed to semitendinosus autografts in rabbit femoral tunnels formed distinct fibrocartilage transition zones at the tendon–bone interface at 12 weeks, with significantly higher staining of BMP-2, VEGF, and BMPR1A compared to titanium screws [11]. Enhanced expression of fibrocartilage markers (Aggrecan, COL2A1, SOX-9) and increased GAG production were observed, with the proposed mechanism being Mg^2+^-stimulated BMP-2 and VEGF accumulation driving fibrochondrogenesis [11]. In a companion study, HP-Mg screws showed substantially superior direct pull-out force at 3 weeks with significantly decreased MMP-13 expression (inhibiting graft degradation) and higher Collagen II expression [79].

**Fibrous tissue mineralization and BMSC recruitment**. Wang et al. (2017) [80] showed that Mg screws significantly promoted mineralization at the tendon graft enthesis and new bone formation in the peri-screw region, with no tunnel widening [80]. A multiscale analysis revealed enhanced recruitment of BMSCs to peri-implant tissue, attributed to upregulation of TGF-β1 and PDGF-BB [81].

**Peri-tunnel bone loss attenuation**. Mg-6Zn-0.5Sr alloy interference screws completely degraded within 12 weeks in rabbit ACLR, with bony ingrowth rapidly filling the cavity by 16 weeks. Peri-tunnel bone loss was significantly attenuated compared to PLA screws, and maximum load to failure was significantly improved at 16 weeks [49].

**Biomechanical performance**. Ex vivo testing of MgYREZr-alloy screws demonstrated maximum pull-out forces of 1001–1092 N, significantly higher than commercial PLGA Milagro screws (787 N, *p* < 0.05) [47]. Cyclic loading (1000 cycles, 0–200 N) showed no significant difference in pull-out force between foam block and cadaveric setups, confirming adequate fixation ability [48].

**Anti-fibrotic effects**. A recent study demonstrated that Mg^2+^ ions upregulate PGE2 production in macrophages, reversing TGF-β1-induced fibroblast-to-myofibroblast trans-differentiation and thereby inhibiting scar tissue formation and promoting a ligament-like phenotype in graft mid-substance with significantly improved gait performance and failure load [82].

**Meta-analysis**. Shang et al. (2021) [77] conducted a meta-analysis of seven animal studies and found that the mechanical properties of the femoral–tendon graft–tibia complex fixed with Mg/alloys were comparable to Ti/alloys, while Mg/alloys were superior to Ti/alloys in promoting new bone formation [77].

### 4.2. ACL Repair: Magnesium Ring Device

An innovative mono-crystalline Mg ring device for primary ACL repair (rather than reconstruction) was tested in cadaveric goat stifle joints. Under 67-N anterior tibial load, the Mg ring repair reduced anterior tibial translation by >50% compared to the ACL-deficient joint at 30°, 60°, and 90° flexion, restoring in situ forces in the ACL and medial meniscus to near-normal levels [83]. Finite element analysis of the same device confirmed a more modest ~50% reduction in AP tibial translation, with in situ force restored to ~60% of intact ACL, reflecting differences between the computational and cadaveric testing methods [84].

### 4.3. Rotator Cuff Repair

Fluoridized ZK60 Mg alloy suture anchors for rotator cuff repair in goats demonstrated new bone formation surpassing the titanium group, with successful tendon healing and stable hematology at 12 weeks [85]. Injectable self-healing hydrogels with sustained release of Mg and curcumin significantly enhanced tendon-to-bone healing in a rat rotator cuff tear model at 8 weeks through anti-inflammatory/antioxidation effects and stem cell chondrogenesis. 3D-printed Mg-incorporated PCL scaffolds (10% Mg optimal) promoted M2 macrophage polarization and inhibited inflammation signaling in rat rotator cuff repair [86,87]. Oral Mg^2+^ supplementation in ovariectomized rats with rotator cuff tear significantly promoted humeral tuberosity bone formation and enthesis regeneration through relieving stem cell replicative senescence [88].

### 4.4. Meniscal Repair

Zhang et al. (2019) [89] demonstrated that HP-Mg stitches for meniscal repair in rabbits (*n* = 120) promoted adhesion and migration of synovial fluid-derived mesenchymal stem cells and stimulated fibrochondrogenesis. At 12 weeks, the Mg group showed enhanced tissue regeneration, less cartilage degeneration, and retained mechanical strength compared to absorbable suture controls [89].

### 4.5. Acromioclavicular Joint and Upper Extremity Applications

No preclinical or clinical data currently exist for Mg fixation in many upper extremity injury and fracture applications, representing an important gap for future investigation. For example, coracoclavicular ligament reconstruction for acromioclavicular joint dislocations currently relies on suture-based cerclage techniques [90], with evolving tunnel-less modifications designed to reduce bone loss and simplify the procedure [3]. Biodegradable Mg interference screws or buttons could provide temporary fixation with osteogenic stimulation at the coracoid and clavicle bone tunnels.

### 4.6. Summary of Molecular Mechanisms

Table 4 summarizes the molecular pathways and mediators through which magnesium ions influence tendon–bone healing and osteogenesis.

## 5. Clinical Translation: Fracture Fixation and Beyond

### 5.1. Commercially Available Devices and Regulatory Status

The MAGNEZIX^®^ compression screw (Syntellix AG, Hannover, Germany), based on the MgYREZr (WE43) alloy, was the first biodegradable Mg orthopedic implant launched to the clinical market in March 2013 with CE mark approval [19]. It is available in 2.7 mm, 3.2 mm, and 4.8 mm diameters [92]. The K-MET screw (U&I Corporation, Uijeongbu, Republic of Korea) received KFDA approval [93]. As of 2025, one FDA-approved Mg-based device exists for orthopedic applications in the United States, with more regulatory approvals in Europe and Asia [10]. ZX00-based screws (Mg-Zn-Ca, rare-earth-free) have entered first-in-humans studies for ankle fracture fixation [7].

### 5.2. Key Clinical Studies

**Hallux valgus (landmark RCT)**. The first prospective RCT (Windhagen et al., 2013 [94]; *n* = 26) randomized patients to MgYREZr or titanium screws for chevron osteotomy fixation. At 6 months, no significant differences were found in AOFAS score, VAS pain, or ROM, with no foreign body reactions, osteolysis, or systemic inflammatory reactions [94]. Three-year follow-up of the same cohort confirmed sustained equivalence across all clinical scores, with Mg implants showing significantly fewer MRI artifacts and no implant-related cysts [95]. A subsequent prospective comparative study (Gazit et al., 2024 [96]; *n* = 60) found significantly faster osteotomy healing in the Mg group, though hardware failure was more common (19.2% vs. 0% for Ti); notably, hardware removal was more common in the Ti group (17.6% vs. 0% for Mg) [96].

**Medial malleolar fractures**. Kose et al. (2018 [97]; *n* = 11) reported mean AOFAS 94.9 ± 5.7 with 100% radiographic union, no complications, and no implant removal or revision at 17.3 months [97]. The ZX00 first-in-humans study (Herber et al., 2022 [7]; *n* = 20) demonstrated that all patients were pain-free at 12 months, with an AOFAS 89.8 ± 7.1 and no Mg screws requiring removal [7]. In the same cohort, at 2.5-year follow-up, implants were radiographically invisible in 17/18 patients [29].

**Scaphoid fractures**. Könneker et al. (2023 [98]; *n* = 12) reported bone healing in all eight acute fractures (100%) and 3/4 nonunions (75%) treated with MAGNEZIX CS, with a Modified Mayo Wrist Score of 95 (fractures) and 80 (nonunions) at ~32 months [98]. Stable fixation of the carpus is paramount in scaphoid fractures, where Mg screw resorption and replacement by bone could eliminate the need for hardware removal in a confined anatomical space.

**Osteonecrosis of the femoral head (RCT)**. Zhao et al. (2016 [99]; *n* = 48) randomized patients to Mg screw fixation of vascularized bone grafts vs. grafting without fixation. At 12 months, HHS was significantly improved in the Mg group with ~25% screw diameter reduction and normal serum Ca, Mg, and P levels [99].

**Displaced femoral neck fractures**. Yu et al. (2015 [100]; *n* = 19 young adults) reported union in 18/19 (94.7%) at a mean of 4.1 months with no avascular necrosis [100].

### 5.3. Pediatric Applications

The largest pediatric series (Stürznickel et al., 2021 [101]; *n* = 89) included fracture stabilization (*n* = 38), osteotomy (*n* = 18), and osteochondral refixation (*n* = 33). Clinical outcomes were rated good to very good in all patients, with revision surgery in only 1/89 (1.1%) and no Mg implants requiring surgical removal [101]. Radiographic analysis of 29 pediatric patients (57 MAGNEZIX screws) confirmed that radiolucent zones were universal but self-limiting, with nearly complete disappearance by ~100 days for 2.7 mm screws and ~200 days for 3.2 mm screws [92].

### 5.4. Meta-Analysis

A systematic review and meta-analysis (Sukotjo et al., 2020 [20]) of 8 studies (468 patients total) found no significant difference in complication rates between Mg and Ti (*p* = 0.868), with an estimated complication rate for Mg screws of 13.3% (95% CI: 8.3–20.6%); a direct comparative analysis was available for 443 of these patients (230 treated with Mg screws and 213 with Ti screws), with the remaining patients in non-comparative single-arm cohorts within the same eight studies [20].

### 5.5. Sports-Relevant Clinical Applications

The pediatric series included 33 osteochondral refixations with good-to-very-good outcomes [101]. A small case series (*n* = 3) of intercondylar eminence fractures, a common sports injury, treated with Mg resorbable screws demonstrated complete screw resorption at 6 months and replacement by newly formed bone at 12 months, with excellent functional recovery [102]. However, no clinical study has yet evaluated Mg interference screws for ACL reconstruction, Mg suture anchors for rotator cuff repair, or Mg devices for meniscal fixation in humans.

### 5.6. Summary of Key Clinical Studies

Table 5 summarizes the key clinical studies of magnesium-based fixation devices.

## 6. Imaging Compatibility

Imaging compatibility is a critical advantage of Mg implants in sports medicine, where serial MRI is essential for monitoring graft maturation, detecting re-tears, and evaluating articular cartilage.

### 6.1. MRI Artifact Comparison

Mg implants produce significantly lower MRI artifacts than titanium across all tested settings. Espiritu et al. (2022) [13] tested in vitro corroded Mg-based screws and Ti equivalents per ASTM F2119, in vivo rat femur implants, and clinical MAGNEZIX^®^ screw data from scaphoid fracture patients, finding that Mg-based implants outperformed Ti in all experiments by producing significantly lower metallic artifact (*p* < 0.05) [13]. Critically, artifact production decreased further as the Mg implant degraded, meaning imaging quality improved over time [13]. In a spinal spacer model, Mg artifact behavior was nearly identical to carbon-fiber-reinforced polymer spacers (*p* > 0.05) and statistically significantly smaller than Ti (*p* < 0.001) [103].

Standard fat suppression techniques (STIR) are not significantly impaired by Mg implants, and metal artifact reduction sequences (SEMAC-VAT, MAVRIC) are not required for adequate imaging [104]. Pile-up artifacts (signal hyperintensity) were present in 100% of MRI examinations, but geometric distortion was absent [104].

### 6.2. MRI Safety

RF-induced heating of non-degraded Mg (WE43) screws was comparable to Ti equivalents, with no statistical difference [105]. As degradation progresses, heating decreases: volume loss and edge smoothing reduced maximum SAR_1_g by up to 12% [106]. Even fractured screws (a late degradation event) remained below IEC safety limits in human voxel models [106].

### 6.3. Hydrogen Gas on Imaging

Hydrogen gas production is a hallmark of Mg degradation with a characteristic imaging appearance. On radiographs, gas in bone increases up to week 5, then gradually decreases, and complete resolution typically occurs by 3 months [107]. On MRI, gas locules were found in bone and soft tissues in 100% of examinations during active resorption, with intra-articular gas in 40% [104].

Gas cavities do not primarily contain hydrogen, despite common assumption. Hydrogen exchanges very rapidly after formation (most within 1 h), and cavities contain only low hydrogen concentrations even shortly after formation [108].

### 6.4. Radiolucent Zones

Radiolucent zones around Mg screws are universal and self-limiting. In 29 pediatric patients with 57 MAGNEZIX implants, radiolucent zones were observed in 93% at first follow-up (6–8 weeks) but significantly decreased from a zone-to-screw area ratio of 2.10 to 1.64 by 12–24 weeks (*p* = 0.0006) [92]. For 2.7 mm screws, zones nearly disappeared by ~100 days, and for 3.2 mm screws by ~200 days [92].

### 6.5. Distinguishing Normal Degradation from Pathology

Normal Mg degradation findings closely mimic infection on all imaging modalities. A prospective MRI evaluation (Waelti et al., 2023 [104]; *n* = 17 pediatric patients, 30 MRIs) found that gas locules (100%), bone marrow edema (100%), soft tissue edema (100%), osteolysis along the screw (87%), periosteal reaction (87%), and joint effusion (50%) were all expected findings during active Mg screw resorption [104]. These should not be misinterpreted as infection. Key differentiating features include the predictable temporal course (gas peaks at ~5 weeks, radiolucent zones follow logarithmic decline), absence of systemic inflammatory signs, peri-implant rather than geographic osteolytic pattern, and self-limiting nature without treatment [92,104,107].

**Figure 3:**
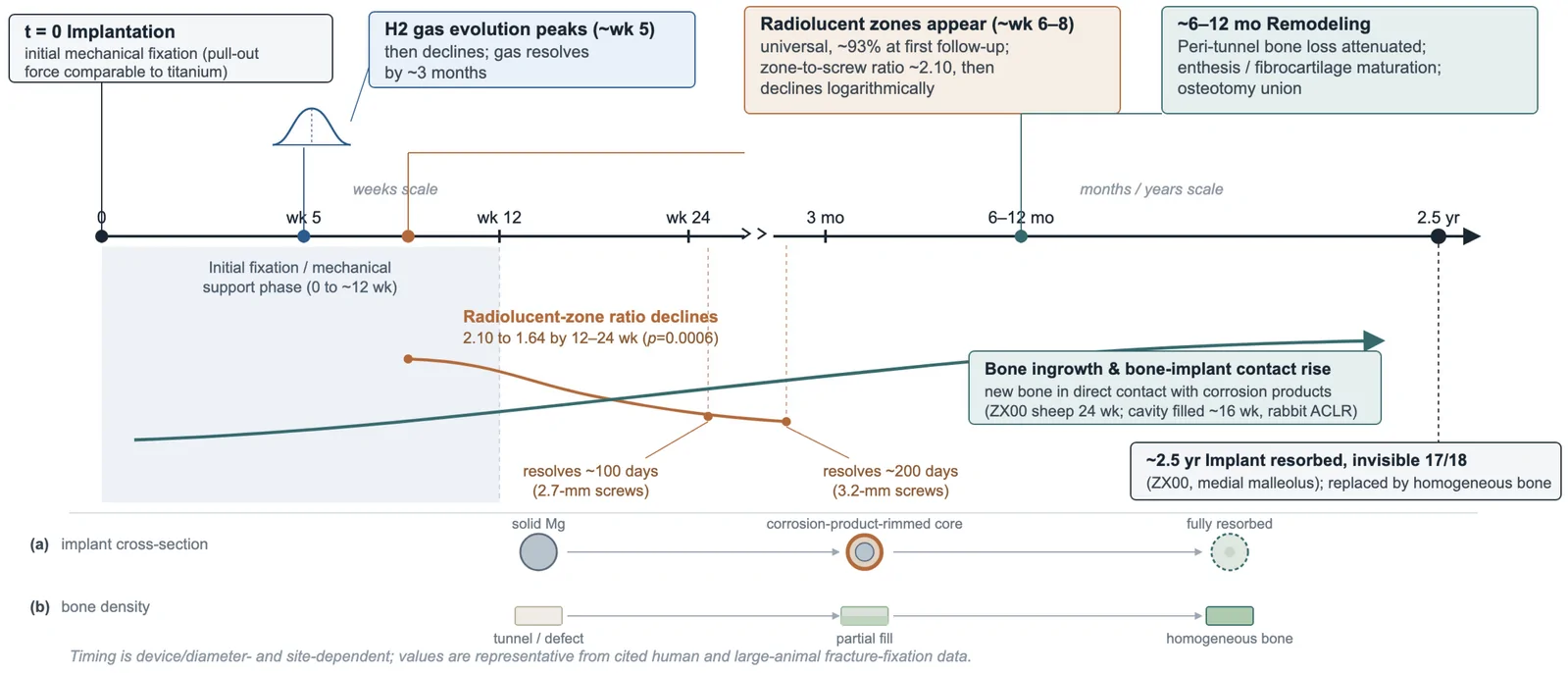
Representative timeline from implantation through degradation to bone regeneration for a magnesium-based orthopedic screw. After implantation, the device provides initial mechanical fixation. Hydrogen-gas evolution peaks near week 5 and resolves by about 3 months; radiolucent zones appear at 6 to 8 weeks (zone-to-screw ratio about 2.10, declining to 1.64 by 12 to 24 weeks) and resolve by about 100 days for 2.7 mm screws and 200 days for 3.2 mm screws. Bone ingrowth and bone–implant contact rise in parallel, and by about 2.5 years the implant is resorbed and radiographically invisible (17 of 18 ZX00 medial malleolar screws). Timing is device-, diameter-, and site-dependent; values are representative of cited human and large-animal fracture-fixation data.

The representative temporal sequence of these degradation and bone-healing events is summarized in Figure 3.

### 6.6. Emerging Imaging Modalities for Tissue–Implant Assessment

Beyond conventional MRI, emerging optical imaging modalities may enhance assessment of tissue–implant interfaces. Second harmonic generation (SHG) imaging has been demonstrated as a label-free, non-destructive method for evaluating collagen architecture in upper extremity nerve injuries in both cadaveric [109] and animal models [110]. SHG imaging can visualize connective tissue disruption and collagen disorganization, allowing visual evaluation of the extent of structural damage and injury severity intraoperatively [110,111]. Adaptation of SHG or similar collagen-sensitive imaging to evaluate tendon graft remodeling and enthesis regeneration around degrading Mg implants represents a promising future research direction [112].

**Figure 4:**
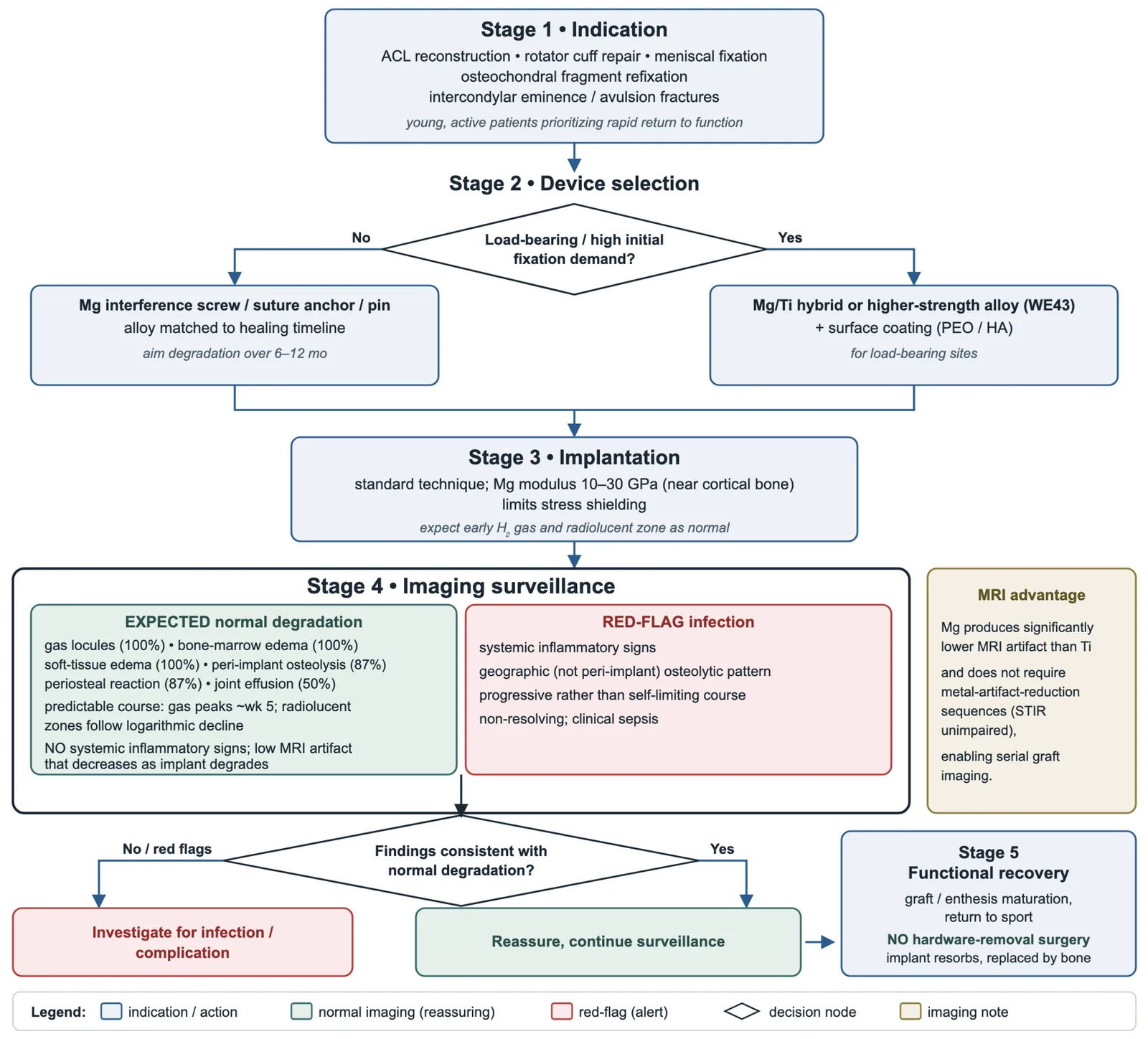
Proposed clinical workflow for magnesium-based implants in sports orthopedic surgery. Candidate indications include ACL reconstruction, rotator-cuff repair, and meniscal and osteochondral fixation. Device selection is guided by the load environment, with Mg/Ti hybrid or higher-strength alloys for high-demand sites. The emphasized step is imaging surveillance, where expected features of normal degradation (gas locules, transient edema, peri-implant radiolucency following a predictable temporal course) are distinguished from red-flag features of infection. Recovery proceeds without hardware-removal surgery because the implant resorbs and is replaced by bone.

A practical clinical workflow that incorporates these imaging considerations is proposed in Figure 4.

## 7. Challenges, Limitations, and Future Directions

### 7.1. Degradation Rate Control

Rapid and non-uniform degradation remains the primary barrier to clinical adoption. Early resorption (<3 months) and inadequate strength are the main challenges hindering use in bone fixation, particularly for load-bearing sports medicine applications [51]. Non-uniform (pitting) corrosion is particularly detrimental, causing abrupt deterioration in mechanical strength [52]. Synchronizing degradation with bone healing remains an unsolved challenge, especially in metabolically compromised patients [5].

### 7.2. Stress Shielding Mitigation

The elastic modulus of Mg alloys (10–30 GPa) closely matches that of cortical bone, significantly reducing stress shielding compared to titanium (~110 GPa) [5,6]. A direct comparison in rats demonstrated that a biodegradable Mg alloy (WE43-based) achieved significantly greater maximum push-out force, ultimate shear strength, and energy absorption compared to titanium controls at 4, 12, and 24 weeks, along with significantly higher bone–implant contact and bone volume per tissue volume [50].

### 7.3. Additive Manufacturing

3D printing of Mg is still in its infancy compared to Ti and CoCr alloys [113]. Laser powder bed fusion (LPBF) is the most studied approach, achieving relative densities >99.5% for WE43 alloy, but AM-Mg scaffolds degrade faster than conventionally manufactured counterparts due to enlarged surface area and microstructural heterogeneity [114,115]. PEO surface modification significantly reduces the degradation rate of AM scaffolds [114]. Extrusion-based/solvent-cast 3D printing offers a room-temperature alternative that avoids the safety hazards of high-energy laser processing of reactive Mg powder, producing scaffolds with hierarchical interconnected pores and degradation rates within the desired range for bone substitution [116,117]. A comparative in vivo study of LPBF-fabricated Ti, Mg, and Zn scaffolds showed that Mg and Zn scaffolds delivered superior therapeutic outcomes compared to Ti scaffolds [118].

### 7.4. Standardization of Corrosion Testing

A fundamental disconnect exists between in vitro and in vivo corrosion rates. Witte et al. (2006) [119] found that in vivo corrosion was approximately four orders of magnitude lower than in vitro corrosion per ASTM norms, with the relative ranking of alloy corrosion rates often reversing between the two settings [119]. The choice of test solution dramatically affects results: EBSS buffered with sodium bicarbonate provides degradation rates comparable to in vivo, while HEPES buffer significantly increases rates [120,121]. Current ASTM standards (F3268-18a, G1-03) and ISO 10993 [122] were not designed specifically for biodegradable metals and have limited value for biocompatibility screening of degradable biomaterials [19,123]. Development of Mg-specific standardized testing protocols remains an urgent need.

### 7.5. Regulatory Pathway

Regulatory complexity arises because Mg acts as both a structural material and a biological agent (pro-osteogenic, anti-osteoclastic, anti-inflammatory), adding an additional regulatory layer beyond standard device evaluation [10]. The gap between existing ISO/ASTM standards and the unique behavior of biodegradable metals necessitates the development of Mg-specific testing standards [19]. The SCAMAG trial, a multicenter, blinded-observer RCT comparing MAGNEZIX Mg-based Herbert screws with titanium Herbert screws for scaphoid fractures (planned *n* ≈ 190), represents the largest ongoing clinical trial and may provide definitive comparative data [124].

### 7.6. Load-Bearing Applications and Hybrid Systems

Mg alone may not provide sufficient mechanical support for stable fracture fixation at load-bearing sites due to rapid early-stage degradation [53]. Innovative Mg/Ti hybrid fixation systems, combining Mg screws with Ti fixators, provided sufficient mechanical support for load-bearing fracture fixation in a rabbit tibia, with 3-fold higher mechanical strength at 12 weeks vs. 6 weeks and enhanced endochondral ossification compared to Ti-only controls [53]. This hybrid approach may be particularly relevant for sports medicine applications where high initial fixation strength is critical.

**Figure 5:**
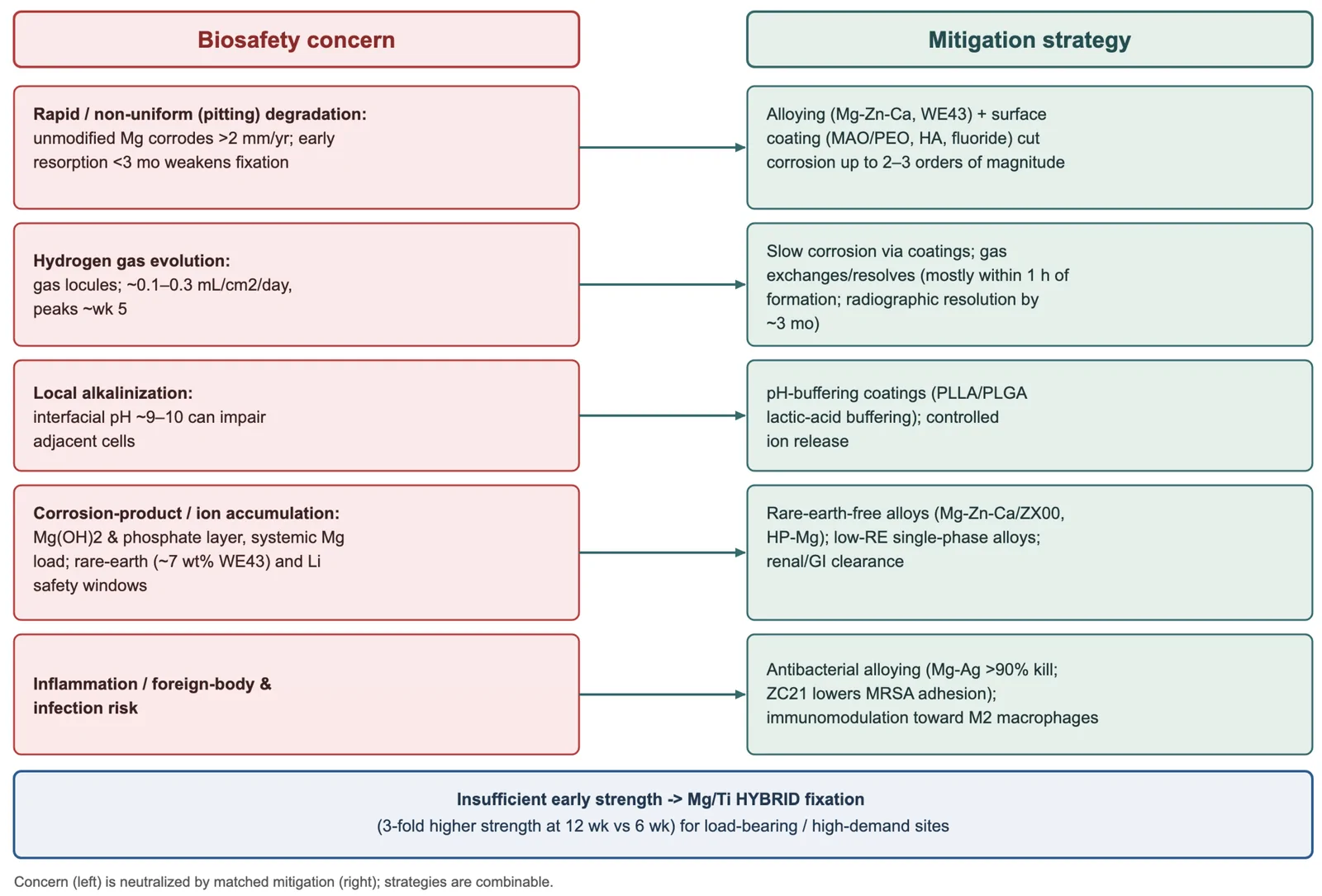
Biosafety concerns of magnesium-based implants paired with mitigation strategies. Each recognized concern of magnesium degradation is matched to an engineering or clinical solution: rapid or non-uniform corrosion is addressed by alloying and surface coating; hydrogen-gas evolution by slower, controlled corrosion; local alkalinization by pH-buffering coatings; corrosion-product and ion accumulation by rare-earth-free or low-rare-earth alloys; and inflammation or infection risk by antibacterial alloying and immunomodulation. Insufficient early mechanical strength at load-bearing or high-demand sites is addressed by Mg/Ti hybrid fixation.

These biosafety considerations, paired with the engineering and clinical strategies that mitigate them, are summarized in Figure 5.

### 7.7. Emerging Technologies

**Drug-eluting Mg implants** enable localized drug delivery from Mg substrates. Antimicrobial peptide-loaded coatings achieved antibacterial rates >85% with sequential osteogenic promotion [125]. PLA/brushite bilayer coatings demonstrated sustained, controlled release of paclitaxel with enhanced osteoinductive potential [126].

**Smart/responsive Mg implants** represent the next frontier. 4D bioprinting of pH-responsive Mg scaffolds shows higher osteogenic differentiation capacity than identical 3D-printed structures [5,127]. Shape memory polymers combined with Mg-based materials enable temperature-responsive shape transformation for personalized bone defect repair [128,129]. Time-programmed ion release coatings (MAO/sodium alginate) enable stage-adaptive immune niches for bone repair [130].

**Biological augmentation strategies** may complement Mg-based fixation. Amniochorionic membrane allografts have demonstrated earlier functional recovery when used as nerve wraps after stretch injury [131,132]. Similarly, pro-regenerative biologic wraps and protective barriers could theoretically be combined with Mg interference screws to enhance the tendon–bone healing microenvironment in ACL reconstruction.

### 7.8. Computational Modeling and Finite Element Analysis

Computational biomechanics has become a practical tool for anticipating how Mg fixation devices behave before an animal or clinical study is undertaken, complementing the empirical corrosion and mechanical data discussed above (Section 2 and Section 7.1). Finite element (FE) analysis, in particular, allows the stress distribution generated by an implant to be compared across materials under identical loading and geometry, isolating the contribution of the elastic modulus mismatch that drives stress shielding (Section 7.2) [133]. A recent FE study of medial malleolar fracture fixation compared screws made of stainless steel, titanium, Mg, and PLGA under identical loads, mapping how the lower-modulus Mg and polymer constructs redistribute peri-implant stress relative to the stiffer metals [134]; this model is directly relevant to the ZX00 medial malleolar clinical experience summarized in Section 5.2. The approach extends from screws to plate constructs: a three-dimensional FE analysis of Mg (WE43 and ZK60) plate-and-screw systems for LeFort I osteotomy fixation examined their biomechanical stability against a titanium reference under physiological loading, characterizing the trade-off between the lower stiffness of the Mg alloys and construct stability [135]. Within sports medicine specifically, the FE analysis of the mono-crystalline Mg ring device for primary ACL repair already discussed in Section 4.2 estimated a roughly 50% reduction in anterior–posterior tibial translation with in situ force restored to about 60% of the intact ACL, values that were more conservative than the paired cadaveric measurements and that illustrate both the utility and the calibration limits of such models [84].

A second and rapidly developing use of computation is the prediction, rather than the mere measurement, of degradation. Continuum-damage FE corrosion models represent the loss of material integrity as an evolving damage field, and early formulations for bioresorbable Mg devices superposed a uniform microgalvanic corrosion term with a stress-dependent stress-corrosion term so that mechanically loaded regions corrode preferentially [136]. Subsequent frameworks implemented corrosion-driven mass loss within implicit FE solvers, improving numerical stability for complex device geometries [137], and the range of phenomenological, continuum-damage, and phase-field strategies has been surveyed for bioabsorbable metal implants [138]. For orthopedic Mg specifically, degradation models have been parameter-identified against experimental data and then validated, so that the corrosion behavior of an implant can be forecast for a given alloy and environment [139]. These methods directly address the in vitro to in vivo disconnect and the absence of Mg-specific test standards noted in Section 7.4: a validated computational model offers a route to interpolate between poorly comparable bench conditions and physiological degradation, although its predictive value is bounded by the quality of the input corrosion parameters.

Computation also enables design optimization rather than post hoc evaluation. Because the same FE machinery that predicts stress concentration can be coupled to the stress-corrosion term described above, implant geometry can be iterated to reduce both peak stress and stress-accelerated corrosion. This was demonstrated for a Mg bone screw whose thread geometry was optimized using FE guidance to lower stress concentration and improve corrosion resistance, with the optimized design subsequently shown to favor fracture healing in vivo [140]. When applied to sports medicine hardware, such geometry- and topology-optimization workflows could, in principle, tailor interference-screw thread forms or suture-anchor profiles to the loading environment of a specific reconstruction, though no such optimized soft-tissue fixation device has yet been reported.

The most integrated direction couples degradation, bone response, and load transfer within a single multiphysics simulation, an approach that begins to resemble a digital twin of the healing construct. A recent model simulated bone growth and mineralization surrounding biodegradable Mg-based implants and compared them with permanent titanium, linking the evolving implant geometry from corrosion to the peri-implant bone that forms and to the resulting load transfer over time [141]. Coupling corrosion, mechanics, and biology in this way is conceptually attractive because it captures the moving target that distinguishes degradable from permanent fixation, but current implementations remain generic rather than patient-specific and are validated against a narrow set of experimental conditions. The unresolved need is therefore for corrosion-coupled, experimentally validated, patient-specific models that ingest individual imaging and loading data and predict the coupled evolution of implant degradation, mechanical support, and bone healing. Developing and validating such models against the large-animal and first-in-humans data called for elsewhere in this review is a concrete priority for future work.

### 7.9. Patient-Specific and Precision-Medicine Approaches

A recurring theme across the alloy, coating, and manufacturing literature is that the optimal Mg implant is unlikely to be a single off-the-shelf geometry applied uniformly across patients. Patient-adapted Mg scaffolds and fixation devices, whose external contour is derived from the individual bone defect on preoperative CT, have been proposed as a route to improved defect filling, load transfer, and osseointegration relative to standardized stock implants [142]. In sports orthopedic surgery this rationale applies to bone tunnels of variable diameter and obliquity in ACL reconstruction, to osteochondral fragments of irregular shape, and to coracoid and clavicular tunnels in coracoclavicular reconstruction (Section 4.5), where a device matched to the host geometry could restore native contact mechanics more faithfully than a fixed-diameter screw or anchor. The concept extends the additive-manufacturing work discussed in Section 7.3 [113,114,115,116,117,118] from generic porous constructs to per-patient geometries, and connects to the shape-memory strategies for personalized bone defect repair noted in Section 7.7 [128,129], in which a temperature-responsive material conforms to a defect intraoperatively rather than being pre-shaped to a population-average template.

Realizing patient-specific Mg devices at clinically acceptable tolerances depends on digital manufacturing routes that can move from an imaging dataset to a finished implant with controlled dimensions and porosity. Two complementary approaches have been reported. Binder jet additive manufacturing followed by automated post-machining has been demonstrated as an end-to-end workflow for personalized Mg implants, combining the geometric freedom of powder-bed printing with subtractive finishing to achieve the dimensional fidelity that fixation hardware requires [143]. Separately, multiscale customized 3D-printed WE43 scaffolds have been produced whose macroscale outline is tailored to CT-derived defect geometry while meso- and nano-scale features generate hierarchical, interconnected porosity, with degradation tempered so that mass loss is better aligned with bone ingrowth [144]. These routes remain subject to the fabrication and quality-control constraints specific to Mg (difficult powder handling, powder splash from the low vaporization temperature, and process-reproducibility issues) already outlined in Section 7.3 [145], so batch-to-batch consistency, not only per-patient design, will govern how quickly customized Mg implants reach the clinic.

Personalization of geometry addresses only part of the problem, because the degradation rate is itself a patient-dependent variable rather than a fixed property of the alloy. The manuscript’s own data illustrate this directly: WE43 wires corroded dramatically faster in hyperlipidemic (ApoE^−^/^−^) mice, with wire breakage in 50% of implants at 10 days versus 0% in wild-type animals [37], and synchronization of degradation with bone healing is described as the hardest to achieve in metabolically compromised patients (Section 7.1) [5]. Taken together, these observations imply that matching resorption to healing cannot be solved by implant geometry alone; the same device may degrade at clinically different rates depending on host lipid status, perfusion, and comorbidity. A precision-medicine framing therefore treats the target degradation profile as an input to be tuned to the individual patient’s physiology, with alloy selection and surface engineering (Section 3) chosen to meet that target. The computational corrosion and bone-remodeling models discussed in Section 7.8 offer the most plausible route to such per-patient tuning, since a validated model that incorporates patient-specific loading and local physiological conditions could, in principle, predict the degradation trajectory for a given geometry before implantation. This capability remains aspirational: current models are calibrated largely against animal and in vitro data, patient-level corrosion predictors are not yet defined, and no prospective study has tested whether individualized Mg implant design improves outcomes over standardized devices, making this a concrete but presently unproven direction for future work.

### 7.10. Barriers to Clinical Translation

The preceding subsections have each examined a single technical obstacle in isolation. Read together, however, they reveal a more fundamental observation: that two decades of encouraging preclinical and fracture-fixation data have not produced a single approved Mg device for soft-tissue-to-bone fixation in sports medicine. The barriers are interdependent rather than additive, and they can be grouped into seven domains that collectively gate clinical translation. The first is regulatory. As set out in Section 7.5, Mg occupies an awkward position between a structural device and a locally acting biological agent, because its degradation products are themselves pro-osteogenic, anti-osteoclastic, and immunomodulatory rather than inert [10]. This dual character places Mg outside the assumptions of the device-approval frameworks written for permanent metals, and the existing ISO 10993 and ASTM standards were not designed for materials that are intended to disappear [19]. The absorbable-metals family as a whole (Mg, Fe, and Zn) still lacks a mature, internationally harmonized standards base, and regulatory expectations differ substantially between Europe, the United States, and Asia, so that a device cleared in one jurisdiction faces a fresh evidentiary burden in the next [146].

A second, less frequently discussed barrier is manufacturing and quality control. Reactive Mg powder is difficult to prepare and handle: its low vapor temperature and high vapor pressure promote powder splash and low usable-powder yield, and the same reactivity that makes Mg attractive biologically creates fire and crack-formation hazards during high-energy laser powder-bed fusion, which is the additive route discussed in Section 7.3 [144]. Beyond additive manufacturing, process-stability and reproducibility problems mean that yield strength and, more importantly, corrosion rate are strongly process-dependent, a point already made for wrought and heat-treated material in Section 2 and Section 7.1. Because thermomechanical history can shift yield strength by a factor of two to three and a corrosion rate by an order of magnitude within a single alloy composition, batch-to-batch property scatter is not a manufacturing detail but a determinant of in vivo degradation behavior, and controlling it is a prerequisite for the tight tolerances a regulator will expect [145]. This manufacturing variability feeds directly into the third barrier, corrosion variability and the in vitro/in vivo disconnect (Section 7.4). In vivo corrosion has been reported to be several orders of magnitude slower than in vitro corrosion measured under ASTM norms, and the relative ranking of alloys can reverse between the two settings, so that bench data cannot yet be relied upon to predict clinical degradation [119]. The choice of test medium and buffer (for example bicarbonate-buffered EBSS versus HEPES) itself changes measured rates by a wide margin [120,121], and the standards currently applied were not written for degradable metals [123]. Without validated, Mg-specific corrosion protocols and the still-incomplete biodegradable-metal standards noted above [19,146], comparisons across studies remain difficult and implant standardization is correspondingly hard to achieve.

The fourth and fifth domains concern value and safety, the two questions a payer and a regulator respectively will ask. The health-economic case for Mg rests almost entirely on avoiding the second, hardware-removal operation that permanent metal frequently requires, a burden that is now documented rather than assumed: in a single-center series, routine implant removal accounted for 3.6% of all orthopedic operations and had a mean operative time of 77.1 min, a mean 4.2-day stay, and a 6% complication rate [147]. Against this avoided cost must be set the higher device and manufacturing cost of Mg, driven by the powder-handling and quality-control demands described above; the net value proposition therefore turns on indications where removal would otherwise be likely (Section 5.2), and it has not been formally established for soft-tissue fixation. Safety of the degradation products is the fifth barrier and remains only partly characterized for the alloys nearest to market. WE43 carries roughly 7 wt.% rare-earth content whose long-term systemic handling, although apparently favorable for low-solubility single-phase Mg-RE systems that are excreted renally and enterally [32], is not established across metabolic phenotypes; Mg-Y-Nd corrosion was dramatically accelerated in a hyperlipidemic model [37], and lithium-bearing systems face the narrow serum therapeutic window that constrains implant volume [41]. Even the local immune response, increasingly understood as a driver of the osteogenic benefit rather than a nuisance, has a double edge: Mg degradation products modulate macrophage polarization and cytokine signaling [148], and the accompanying alkalinization, hydrogen evolution, and particulate release can engage inflammasome and P2X7/NLRP3 pathways in the peri-implant microenvironment [149]. Beneficial IL-10-dependent M2 polarization has been shown to promote subperiosteal osteogenesis [150], but the same work indicates that a degradation rate that is too fast can convert a transient, pro-osteogenic inflammatory phase into persistent low-grade marrow inflammation with adipogenesis [151], which again ties safety back to the unresolved problem of degradation control (Section 7.1).

The seventh and arguably decisive barrier is the near-absence of large, multicenter randomized evidence. The clinical dataset summarized in Section 5 is dominated by small single-arm cohorts and a handful of modest RCTs in fracture fixation, and for soft-tissue reconstruction in sports medicine there is at present no human trial at all (Section 8). The SCAMAG trial of Mg versus titanium Herbert screws for scaphoid fractures (planned n, approximately 190) is the notable exception and the largest ongoing comparative study [124], but a single fracture-fixation RCT cannot answer the enthesis-healing questions that matter for ACL, rotator cuff, or meniscal applications. Compounding the scarcity is the statistical fragility of the orthopedic trial literature more broadly: systematic fragility analyses across several orthopedic domains report that reversing the outcome of only a small number of patients would overturn the primary result, with median fragility indices typically between 1 and 5 [152,153,154,155]. Much sports-medicine bioinductive-implant evidence is moreover Level IV and frequently uncontrolled [156], so the apparent equivalence of Mg to titanium rests on a thinner statistical base than the headline complication rates suggest. These fragility data are analogous rather than direct evidence, drawn from adjacent subspecialties rather than from Mg trials (Section 8), but they set a clear standard: adequately powered, methodologically rigorous multicenter RCTs, not further underpowered case series, are what will move Mg fixation from preclinical promise to routine sports-orthopedic practice [157]. Table 6 consolidates the principal advantages and countervailing limitations across the domains that determine this trajectory.

## 8. The Translational Gap: From Bench to Sports Medicine Bedside

**Figure 6:**
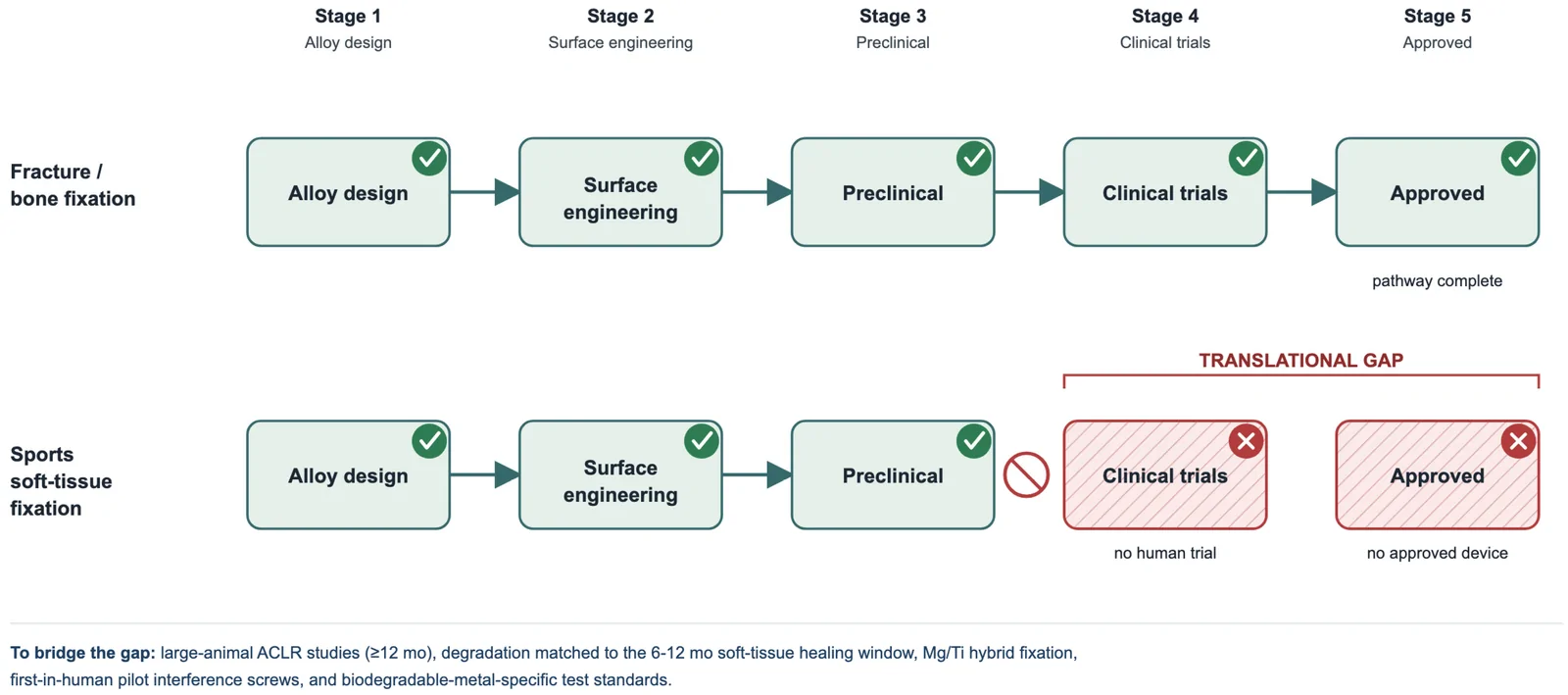
Translational pathway for magnesium-based orthopedic implants and the sports-medicine gap. Development proceeds from alloy design through surface engineering, preclinical testing, and clinical study to an approved device. For fracture and osteotomy fixation this pathway is complete (CE-marked, KFDA- and FDA-approved devices; more than 468 patients with complication rates equivalent to titanium). For sports soft-tissue indications (ACL, rotator cuff, meniscus), strong preclinical data exist, but no human trial has been performed and no device is approved, defining the translational gap that the proposed bridging steps are designed to close.

This development pathway and the stage at which it currently stalls for sports-medicine indications are depicted in Figure 6.

The evidence reviewed above reveals a striking translational gap. On one hand, preclinical data consistently demonstrate that Mg-based interference screws promote fibrocartilaginous enthesis regeneration, attenuate peri-tunnel bone loss, achieve biomechanical fixation comparable to titanium, and recruit endogenous stem cells through multiple molecular pathways [11,49,77,78,80]. On the other hand, no human clinical trial has evaluated Mg fixation devices for any soft-tissue reconstruction in sports medicine.

Nevertheless, the accumulated fracture-fixation experience does more than establish that Mg devices can be implanted safely; taken together, several strands of that evidence lower the threshold for a first-in-human sports-medicine study. First, the pooled safety and equivalence record across more than 468 patients, with complication rates not significantly different from titanium [20], indicates that the systemic and local tolerability of degrading Mg screws is no longer the principal unknown. Second, the now-characterized imaging signature of normal degradation (gas locules, peri-implant radiolucent zones, and transient edema following a predictable temporal course, Section 6) provides the interpretive framework needed to distinguish expected resorption from true complication during postoperative surveillance, which is a prerequisite for running a sports-medicine trial that relies on serial MRI of the graft. Third, the biomechanical parity data, in which Mg and Mg-alloy interference screws match or exceed the fixation strength of polymeric and titanium comparators under pull-out and cyclic loading (Section 4.1), suggest that initial fixation would not be the limiting factor. Collectively, these lines of evidence de-risk, without eliminating, the uncertainty of a first sports-medicine application. Important differences remain that fracture data cannot fully address: soft-tissue-to-bone healing follows a slower and biologically distinct timeline than diaphyseal or metaphyseal union, the intra-articular environment exposes the degrading implant and any evolved hydrogen to synovial fluid and joint motion rather than a contained osseous bed, and graft-tunnel biology (tendon-to-bone integration, tunnel widening, and peri-tunnel bone remodeling) has no direct counterpart in the fracture-fixation literature. These residual gaps define what a first-in-human sports-medicine trial would still need to demonstrate, rather than reasons to withhold one.

Several factors contribute to this gap:

1. **Degradation rate mismatch**. Whether Mg degradation kinetics match the rate of bone–tendon integration remains unresolved. In rabbit ACLR models, Mg-6Zn-0.5Sr screws completely degraded within 12 weeks, potentially too fast for human ACL graft incorporation, which requires 6–12 months [49,158].
2. **Mechanical strength concerns**. Fast degradation and insufficient mechanical strength of pure Mg remain major translational barriers, though alloying (Mg-Zn-Sr, WE43) and surface modification (PEO, fluoride coating) substantially improve both parameters [157].
3. **Animal model limitations**. The preclinical evidence is predominantly from rabbit models (small animals); only one sheep study and limited goat data exist. No large-animal ACLR model with long-term functional outcomes has been reported [77,159].
4. **Regulatory complexity**. The dual nature of Mg as both a structural device and a biological agent complicates the regulatory pathway [10].
5. **Evidence quality and statistical fragility**. A scoping review of 145 studies (6043 patients) found that 65.5% of bioactive/bioinductive implant studies in sports medicine were Level IV evidence, and only 24.8% included a control group [156]. Moreover, even existing orthopedic RCTs may overstate the robustness of their conclusions. Systematic reviews of statistical fragility across multiple orthopedic domains have demonstrated that RCTs evaluating surgical approaches and orthopedic interventions are statistically fragile [152,153,154], meaning that changing the outcome of only a small number of patients would reverse the statistical significance of the primary finding.
6. **Evidentiary caveat**. These fragility estimates are drawn from adjacent orthopedic subspecialties (e.g., carpometacarpal arthritis, general trauma, lumbar disk arthroplasty) rather than from magnesium-implant trials specifically; they are analogous, not direct, evidence intended to motivate adequately powered future Mg-implant studies. The median fragility index across orthopedic RCTs is consistently low, typically ranging from 1 to 5 [152,153,154,155]. These fragility analyses underscore the need for adequately powered, methodologically rigorous RCTs when evaluating Mg-based implants against established fixation methods, particularly given the novelty of the technology and the regulatory scrutiny it will face [155,156,157].

To bridge this gap, the following research priorities are proposed:

- Large-animal ACLR studies (sheep, goat, or canine) with surface-modified Mg interference screws and long-term functional outcomes (≥12 months).
- Development of Mg alloys with degradation rates optimized for soft-tissue-to-bone healing timelines (6–12 months).
- Mg/Ti hybrid fixation systems for ACL reconstruction, combining Mg’s bioactivity with Ti’s initial mechanical strength.
- First-in-humans pilot studies of Mg interference screws for ACL reconstruction, building on the safety profile established in fracture fixation.
- Standardized preclinical testing protocols specific to biodegradable metals in soft-tissue fixation applications.

## 9. Conclusions

Several conclusions can now be drawn with reasonable confidence. The elastic modulus of Mg alloys (10 to 30 GPa) sits within the range of cortical bone, and a direct rat comparison confirmed greater push-out force, shear strength, and bone–implant contact for a WE43-based screw than for titanium at 4, 12, and 24 weeks; thus, the reduction in stress shielding relative to titanium (~110 GPa) is well supported [5,6,50]. For fracture fixation, pooled data from over 468 patients, including a meta-analysis showing no difference in complication rates against titanium (*p* = 0.868), together with the routine elimination of a second removal operation, establish an acceptable clinical safety profile in that setting [20]. Imaging behavior is likewise consistent: Mg implants generate significantly lower MRI artifact than titanium across in vitro, animal, and clinical testing, with artifact decreasing further as the implant degrades [13,103]. At the preclinical level, the enthesis and osteogenic advantages of Mg-based fixation are reproducible across ACL reconstruction, rotator cuff, and meniscal models, spanning fibrocartilage transition-zone formation, attenuated peri-tunnel bone loss, and improved mechanical outcomes [11,49,77,80,89]. Finally, surface engineering is now a dependable lever, with composite and multilayer coatings capable of lowering corrosion rates by two to three orders of magnitude while retaining or enhancing bioactivity [5,73,74].

What remains unresolved is more consequential for translation. No human trial has yet tested a Mg device for any soft-tissue reconstruction in sports medicine, and the central materials question, whether degradation can be synchronized with the 6- to 12-month timescale of tendon-to-bone and graft incorporation, is unsettled; Mg-6Zn-0.5Sr screws degraded completely within 12 weeks in rabbit ACLR, and this synchronization problem is aggravated in metabolic comorbidity, as illustrated by markedly accelerated WE43 corrosion in hyperlipidemic mice [37,49,158]. Standardized, degradable-metal-specific test methods are still lacking, since conventional device standards were not designed for materials that corrode and remodel in situ [19,146]. Large-animal ACLR data with long-term functional endpoints are scarce, and no adequately powered multicenter randomized trial has been reported; the ongoing scaphoid-fracture SCAMAG trial (planned n ~190) is the closest comparator dataset in fracture fixation rather than soft-tissue repair [77,124,159]. Longer-term questions of degradation-product handling and the local immune-inflammatory response also remain open, including the balance between beneficial macrophage-mediated osteopromotion and the persistent low-grade marrow inflammation that faster degradation can provoke [148,149,150,151].

A prioritized path forward follows from these gaps rather than from optimism about the technology. The first requirement is degradation tuned to soft-tissue healing timelines, delivered through surface-engineered devices and, where early fixation strength is limiting, Mg/Ti hybrid interference screws that pair Mg bioactivity with a titanium load path. Computational and patient-specific design can shorten this iteration, with finite-element geometry optimization and imaging-derived, patient-adapted scaffolds allowing screw thread and construct geometry to be matched to the defect anatomy before fabrication [140,142]. These constructs then require validation in large-animal ACLR models with endpoints of at least twelve months, and only afterward should staged first-in-human sports-medicine studies proceed, deliberately built on the fracture-fixation safety base already documented for Mg fixation [20]. Progress is plausible on this route, but the soft-tissue evidence base has to be built from large-animal work upward before Mg devices can be recommended for ACL, rotator cuff, or meniscal fixation in patients.

## Figures and Tables

**Table 1:** Comparison of the principal biomaterial classes used for fixation in orthopedic and sports orthopedic surgery. Elastic modulus values are approximate and, for metals and composites, refer to the bulk material; cortical bone (~15 to 30 GPa) is given as the reference target for modulus matching. Cells summarize representative rather than exhaustive properties, and mechanical values for magnesium and polymers are strongly dependent on processing and composition (see Section 2 and Section 7).

Material Class	Representative Examples	Elastic Modulus (GPa)	Degradation/Resorption	MRI/CT Compatibility	Osteogenic Bioactivity	Revision (Hardware Removal)	Key Limitations/Complications	Clinical Maturity	Ref(s)
Permanent metals	Titanium (Ti-6Al-4V), stainless steel	~110 (Ti), well above bone	Non-degradable; permanent	Substantial MRI artifact; CT beam hardening	Bioinert; osseointegrates but not osteoinductive	Frequently required; second surgery with own morbidity and cost	Stress shielding from modulus mismatch; imaging artifact; reoperation for removal	Established, gold standard	[5,6,7,9]
Biodegradable polymers	PLLA, PGA, PLGA (+/− HA)	Low (well below bone)	Hydrolytic resorption; often slow/incomplete	Radiolucent; low artifact but poor visualization	Bioinert to weakly conductive; not osteoinductive	Avoided in principle; resorbable	Acidic by-products, sterile osteolysis, foreign-body reaction, cyst formation, incomplete ossification	Established (screws, anchors)	[8]
Magnesium alloys	WE43 (MAGNEZIX), ZX00, HP-Mg	10–30, close to bone	In vivo corrosion; H_2_ gas; rate control still a challenge	Lower MRI artifact than Ti; artifact falls as implant degrades	Osteoinductive via Mg^2+^ (BMP-2, VEGF, Wnt/beta-catenin)	Not required; resorbed and replaced by bone	Rapid/non-uniform early corrosion, H_2_ gas, radiolucent zones, strength loss at load-bearing sites	CE-marked (fracture); no sports-medicine trials	[5,6,10,11,12,13,14]
Bioactive ceramics/glass	HA, beta-TCP, 45S5 bioactive glass	High stiffness but brittle	Slow dissolution (composition-dependent)	Radiopaque; generally low metallic artifact	Osteoconductive and osteostimulative via ionic dissolution	Not applicable (graft substitute, not hardware)	Brittle, low fracture toughness; not suited to load-bearing fixation	Established as bone-graft substitute; non-load-bearing	[15,16]
Composite fixation	CFR-PEEK plates/screws	~16–24, near bone	Non-degradable; permanent	Radiolucent; low CT/MRI artifact	Bioinert; not osteogenic	Not resorbable; removal possible if needed	Permanent implant; no biological stimulation; limited long-term data vs. metal	Established in extremity trauma	[17,18]

**Table 2:** Comparative overview of the principal magnesium alloy systems investigated for orthopedic fixation. YS = yield strength; UTS = ultimate tensile strength; RE = rare earth; n/a = not applicable. Mechanical properties are processing-dependent, and the listed ranges represent optimized conditions; direct cross-system comparison requires specification of the thermomechanical processing state.

Alloy System	Representative Compositions	YS (MPa)	UTS (MPa)	Corrosion Rate (mm/yr)	Key Advantages	Clinical Status	Ref(s)
Mg-Zn-Ca	ZX00, ZX10, ZXT100	121–345	~226	0.06–0.21	RE-free; lowest corrosion; osteogenic	First-in-human (ankle)	[7,25,26,27,28,29]
Mg-Y-RE-Zr (WE43)	Mg-4Y-3RE-0.5Zr	NR	300–347	0.16	Highest clinical evidence; CE-marked	CE-marked (MAGNEZIX)	[22,30,33,34,35]
High-Purity Mg	99.9–99.99% Mg	Lower	Lower	0.11–0.40	No alloying toxicity; excellent osteogenesis	Clinical use (China)	[38,39]
Mg-Li	Mg-xLi-Zn, Mg-Li-Al-Zn	193–270	108–208	0.13–0.67	Exceptional ductility (>40%)	Preclinical (stents)	[41,42,43,44]
Mg-Ag	Mg2Ag, Mg4Ag, Mg-Ag-Y	n/a	Up to 325 (comp.)	0.91 (in vivo)	Intrinsic antibacterial (>90% kill)	Preclinical	[45,46]

**Table 3:** Summary of surface coating strategies for magnesium implants, with representative corrosion-rate reduction, principal biological effects, and characteristic limitations.

Coating Strategy	Corrosion-Rate Reduction	Key Biological Effects	Limitations	Ref(s)
MAO/PEO alone	~60% (to 0.3–0.8 mm/yr)	Enhanced bone formation; no cytotoxicity	Inherent porosity requires sealing	[5,55,56]
HA/CaP	To ~0.25 mm/yr	Best in vivo osseointegration	Variable coating adhesion	[5,61,62]
MgF2 conversion	0.49 mm/yr (initial) to 0.01 mm/yr (after 24 h)	BMP-2/Col I upregulation; biofilm inhibition	Short-term effect (~4 weeks)	[64,65,66]
Polymer (PLA/PLGA/PCL)	1–3 orders of magnitude	Drug delivery capability; pH buffering	Delamination risk	[69,70]
Composite (MAO + polymer/CaP)	2–3 orders of magnitude	Synergistic corrosion control + bioactivity	Fabrication complexity	[73,74,75]

**Table 4:** Molecular pathways and mediators through which magnesium ions influence tendon–bone healing and osteogenesis, with the corresponding experimental application models.

Pathway/Molecule	Effect of Mg^2+^	Application Model	Ref(s)
BMP-2/VEGF/BMPR1A	Upregulated, promotes fibrocartilage regeneration	ACL reconstruction (rabbit)	[11]
TGF-beta1/PDGF-BB	Upregulated, promotes BMSC recruitment	ACL reconstruction (rabbit)	[81]
MMP-13	Downregulated, inhibits graft degradation	ACL reconstruction (rabbit)	[79]
Collagen II/Aggrecan/SOX-9	Upregulated, fibrocartilage markers	ACL reconstruction (rabbit)	[11]
CGRP/Periostin	Upregulated, promotes osteogenic differentiation	ACL reconstruction (rabbit)	[91]
PGE2 (macrophage-derived)	Reversed fibroblast-to-myofibroblast transition	ACL reconstruction (rat)	[82]
Canonical Wnt/beta-catenin	Activated, promotes BMSC osteogenic differentiation	Fracture healing (rabbit)	[12]
M2 macrophage polarization	Enhanced, anti-inflammatory microenvironment	Rotator cuff repair (rat)	[87]

**Table 5:** Key clinical studies of magnesium-based fixation devices, ordered by anatomical indication. FIH = first-in-humans; RCT = randomized controlled trial; AOFAS = American Orthopaedic Foot and Ankle Society score; HHS = Harris Hip Score; ONFH = osteonecrosis of the femoral head; n/a = not applicable.

Study	Device/Alloy	Indication	*n*	Design	Follow-Up	Key Outcome	Ref(s)
Windhagen 2013	MAGNEZIX (WE43)	Hallux valgus	26	RCT	6 mo	Equivalent to Ti	[94]
Plaass 2018	MAGNEZIX	Hallux valgus	26	RCT	3 yr	Equivalent; fewer MRI artifacts	[95]
Gazit 2024	Mg screw	Hallux valgus	60	Prospective	1 yr	Faster healing; 19.2% hardware failure; hardware removal 0% (Mg) vs. 17.6% (Ti)	[96]
Kose 2018	MAGNEZIX	Medial malleolus	11	Retrospective	17 mo	AOFAS 94.9; 100% union	[97]
Herber 2022	ZX00 (MgZnCa)	Medial malleolus	20	Prospective FIH	12 mo	AOFAS 89.8; no removal needed	[7]
Labmayr 2024	ZX00	Medial malleolus	18	Prospective FIH	2.5 yr	Implants invisible 17/18	[29]
Konneker 2023	MAGNEZIX CS	Scaphoid fractures	12	Case series	32 mo	100% union (acute)	[98]
Zhao 2016	Pure Mg	ONFH	48	RCT	12 mo	HHS improved vs. control	[99]
Yu 2015	Pure Mg	Femoral neck	19	Prospective	4.1 mo	94.7% union	[100]
Sturznickel 2021	MAGNEZIX (mixed)	Pediatric	89	Retrospective	8.2 mo	Good-very good; 1.1% revision	[101]
Sukotjo 2020	Various Mg	Mixed fracture	468	Meta-analysis	n/a	No difference vs. Ti (*p* = 0.868)	[20]

**Table 6:** Principal advantages and limitations of magnesium-based fixation devices, organized by translational domain. Cross-references indicate the manuscript sections where each item is developed in detail.

Domain	Advantage	Limitation/Open Issue	Ref(s)
Mechanical/elastic modulus	Elastic modulus (10–30 GPa) closely matches cortical bone, reducing stress shielding relative to titanium (~110 GPa)	Lower absolute strength than titanium and rapid early-stage strength loss at load-bearing sites; property scatter is strongly process-dependent	[5,6,50,53,145]
Degradation/resorption	Degrades in vivo to Mg^2+^ with no permanent implant and no need for hardware removal	Degradation is often too fast or non-uniform (pitting), and in vitro rates poorly predict in vivo behavior under non-standardized testing	[49,51,52,119,120,121,123]
Osteogenic bioactivity	Mg^2+^ and degradation products are pro-osteogenic and immunomodulatory (M2/IL-10 polarization), enhancing enthesis and bone formation	Excessively fast degradation can shift a beneficial transient inflammatory phase toward persistent low-grade marrow inflammation and adipogenesis	[11,12,148,150,151]
Imaging	Substantially fewer MRI artifacts than titanium, decreasing further as the implant resorbs; RF heating comparable to or below titanium	Hydrogen gas and radiolucent zones during active resorption can mimic infection and complicate radiographic interpretation	[13,92,104,107]
Infection	Certain systems (Mg-Ag, ZC21, fluoride coatings) show intrinsic antibacterial activity	Antibacterial Mg systems remain preclinical; no clinical infection-outcome data for soft-tissue fixation	[26,45,46]
Reoperation	Avoids the second hardware-removal operation, whose operative time, stay, and complication burden are documented	Value proposition depends on indications where removal is otherwise likely, and is offset by higher device/manufacturing cost	[7,96,144,146]
Regulatory/evidence	One FDA-approved and several CE-marked/KFDA-approved fracture devices; SCAMAG RCT ongoing	Dual device/biological-agent nature and immature biodegradable-metal standards; no sports-medicine RCT; adjacent orthopedic RCTs are statistically fragile	[10,19,124,146,152,153,154,155,156,157]

## Data Availability

No new data were created or analyzed in this study. Data sharing is not applicable to this article.

## Abbreviations

Abbreviation	Definition
3D	Three-dimensional
4D	Four-dimensional
AC	Alternating current (as in AC-MAO processing)
AC joint	Acromioclavicular joint
ACL	Anterior cruciate ligament
ACLR	Anterior cruciate ligament reconstruction
ALP	Alkaline phosphatase
AM	Additive manufacturing
AOFAS	American Orthopaedic Foot and Ankle Society (score)
AP	Anteroposterior (anterior tibial translation)
ApoE	Apolipoprotein E (ApoE^−^/^−^ = apolipoprotein E knockout mice)
ASTM	American Society for Testing and Materials
AZ31B	Magnesium alloy (Mg-3Al-1Zn, grade B)
BCC	Body-centered cubic (crystal structure)
BMP-2	Bone morphogenetic protein-2
BMPR1A	Bone morphogenetic protein receptor type 1A
BMSC	Bone marrow stromal cell (bone marrow-derived mesenchymal stem cell)
Ca(OH)2	Calcium hydroxide
CaP	Calcium phosphate
CC	Compression screw (as in MAGNEZIX CS)
CC ligament	Coracoclavicular ligament
CD	Cyclodextrin (as in γ-CD, gamma-cyclodextrin)
CE mark	Conformite Europeenne mark (European conformity marking)
CGRP	Calcitonin gene-related peptide
CI	Confidence interval
CoCr	Cobalt-chromium (alloy)
Col I/Collagen I	Collagen type I
COL2A1/Collagen II	Collagen type II (alpha 1 chain)
CS	Compression screw (MAGNEZIX CS)
EBSS	Earle’s balanced salt solution
ECAP	Equal-channel angular pressing
FDA	Food and Drug Administration (United States)
FHA	Fluoridated hydroxyapatite
FIH	First-in-humans
GAG	Glycosaminoglycan
GPa	Gigapascal
HA	Hydroxyapatite
HCP	Hexagonal close-packed (crystal structure)
HEPES	4-(2-hydroxyethyl)-1-piperazineethanesulfonic acid (buffer)
HHS	Harris Hip Score
HP-Mg	High-purity magnesium
IEC	International Electrotechnical Commission
ImH	Imidazole (as in Chitosan-ImH@γ-CD coating)
ISO	International Organization for Standardization
K-MET	Commercial magnesium bone screw (U&I Corporation, Uijeongbu, Republic of Korea)
KFDA	Korea Food and Drug Administration
LAE442	Magnesium alloy (Mg-4Li-4Al-2RE)
Li2CO3	Lithium carbonate
LPBF	Laser powder bed fusion
LZ61/LZ91	Mg-Li-Zn ternary alloys (nominal compositions)
M2 macrophage	Anti-inflammatory (alternatively activated) macrophage phenotype
MAGNEZIX	Commercial biodegradable magnesium compression screw (Syntellix AG, Hannover, Germany)
MAO	Micro-arc oxidation
MAVRIC	Multi-acquisition variable-resonance image combination (metal artifact reduction MRI sequence)
mEq/L	Milliequivalents per liter
Mg	Magnesium
Mg(OH)2	Magnesium hydroxide
Mg^2+^	Magnesium ion
Mg3(PO4)2	Magnesium phosphate
MgF2	Magnesium fluoride
MgO	Magnesium oxide
MgSiO3	Magnesium silicate (enstatite)
MgYREZr	Magnesium-yttrium-rare earth-zirconium alloy (WE43)
MMP-13	Matrix metalloproteinase-13
MPa	Megapascal
MRI	Magnetic resonance imaging
mRNA	Messenger ribonucleic acid
MRSA	Methicillin-resistant Staphylococcus aureus
NIR	Near-infrared
NR	Not reported
ONFH	Osteonecrosis of the femoral head
OPN	Osteopontin
OVX	Ovariectomized
PCL	Polycaprolactone
PDGF-BB	Platelet-derived growth factor BB (homodimer)
PEO	Plasma electrolytic oxidation
PGA	Polyglycolic acid
PGE2	Prostaglandin E2
PLA	Polylactic acid
PLGA	Poly(lactic-co-glycolic acid)
PLLA	Poly-L-lactic acid
RCT	Randomized controlled trial
RE	Rare earth (rare-earth element)
RF	Radiofrequency
ROM	Range of motion
RUNX2	Runt-related transcription factor 2
S. aureus	Staphylococcus aureus
S. epidermidis	Staphylococcus epidermidis
SA	Sodium alginate (as in MAO/SA composite coating)
SAR	Specific absorption rate (SAR1g = SAR averaged over 1 g of tissue)
SCAMAG	SCAphoid fracture osteosynthesis by MAGnesium-based headless Herbert screws (clinical trial)
SEMAC-VAT	Slice-encoding for metal artifact correction with view-angle tilting (metal artifact reduction MRI sequence)
SHG	Second harmonic generation (imaging)
SOX-9	SRY-box transcription factor 9
STIR	Short tau inversion recovery (fat suppression MRI technique)
T5	Heat treatment designation (artificial aging after cooling from an elevated-temperature shaping process)
TGF-beta1	Transforming growth factor beta 1
Ti	Titanium
U.S.	United States
UTS	Ultimate tensile strength
VAS	Visual analog scale (pain)
VEGF	Vascular endothelial growth factor
WE43	Magnesium alloy Mg-4Y-3RE-0.5Zr (yttrium-rare earth-zirconium)
Wnt/beta-catenin	Wnt/beta-catenin signaling pathway (canonical Wnt signaling)
wt.%	Weight percent
YS	Yield strength
ZC21	Magnesium alloy Mg-2Zn-0.5Ca (wt.%)
ZK60	Magnesium alloy (Mg-6Zn-0.5Zr)
ZM21	Magnesium alloy (Mg-2Zn-1Mn)
ZX00	Ultra-lean magnesium alloy Mg-0.45Zn-0.45Ca (wt.%), rare-earth-free
ZX10	Magnesium-zinc-calcium alloy (Mg-Zn-Ca family, ~1 wt.% Zn)
ZXT100	Magnesium alloy Mg-1.2Zn-0.32Ca-0.25Sn (wt.%)

## References

[1] Ramey M.D., Swaminathan S.J., Locke A.R., Koehne N.H., Schroen C.A., Swaminathan V.G., Corvi J.J., Capotosto S., Cagle P.J., Hausman M.R. (2025). Trends in Sports-Related Upper Extremity Injuries Presenting to United States Emergency Departments: A Retrospective Analysis of National Injury Data. J. Clin. Med..

[2] Ramey M.D., Locke A.R., Koehne N.H., Schroen C.A., Siniakowicz C., Kim J., Hausman M. (2026). Rising Rates of Sports-Related Hand and Wrist Injuries: An Age- and Sex-Specific Analysis of National Injury Data from 2020 to 2024. Hand Surg. Rehabil..

[3] Ranson W.A., Capotosto S., Schroen C.A., Burapachaisri A., Cagle P.J. (2025). Tunnel-Less Modification of the All-Suture Tape Cerclage Technique for Reconstruction of the Coracoclavicular Ligament Complex. Arthrosc. Tech..

[4] Elahinia M., Ibrahim H., Mahtabi M.J., Mehrabi R. (2019). Engineering Bone-Implant Materials. Bioengineering.

[5] Chaudhari Y.S., Chaudhari M.Y., Gholap A.D., Alam M.I., Khalid M., Webster T.J., Gowri S., Faiyazuddin M. (2025). Surface Engineering of Nano Magnesium Alloys for Orthopedic Implants: A Systematic Review of Strategies to Mitigate Corrosion and Promote Bone Regeneration. Front. Bioeng. Biotechnol..

[6] Staiger M.P., Pietak A.M., Huadmai J., Dias G. (2006). Magnesium and Its Alloys as Orthopedic Biomaterials: A Review. Biomaterials.

[7] Herber V., Labmayr V., Sommer N.G., Marek R., Wittig U., Leithner A., Seibert F., Holweg P. (2022). Can Hardware Removal Be Avoided Using Bioresorbable Mg-Zn-ca Screws after Medial Malleolar Fracture Fixation? Mid-Term Results of a First-in-Human Study. Injury.

[8] Sundaraj K., Salmon L.J., Heath E.L., Winalski C.S., Colak C., Vasanji A., Roe J.P., Pinczewski L.A. (2020). Bioabsorbable versus Titanium Screws in Anterior Cruciate Ligament Reconstruction Using Hamstring Autograft: A Prospective, Randomized Controlled Trial with 13-Year Follow-Up. Am. J. Sports Med..

[9] Abd-Elaziem W., Darwish M.A., Hamada A., Daoush W.M. (2024). Titanium-Based Alloys and Composites for Orthopedic Implants Applications: A Comprehensive Review. Mater. Des..

[10] Lacin N., Sfeir C. (2026). Magnesium-Based Resorbable Biomaterials: Biological Effects to Clinical Use. J. Dent. Res..

[11] Cheng P., Han P., Zhao C., Zhang S., Wu H., Ni J., Hou P., Zhang Y., Liu J., Xu H. (2016). High-Purity Magnesium Interference Screws Promote Fibrocartilaginous Entheses Regeneration in the Anterior Cruciate Ligament Reconstruction Rabbit Model via Accumulation of BMP-2 and VEGF. Biomaterials.

[12] Hung C.-C., Chaya A., Liu K., Verdelis K., Sfeir C. (2019). The Role of Magnesium Ions in Bone Regeneration Involves the Canonical Wnt Signaling Pathway. Acta Biomater..

[13] Espiritu J., Berangi M., Yiannakou C., Silva E., Francischello R., Kuehne A., Niendorf T., Könneker S., Willumeit-Römer R., Seitz J.-M. (2022). Evaluating Metallic Artefact of Biodegradable Magnesium-Based Implants in Magnetic Resonance Imaging. Bioact. Mater..

[14] Ibrahim H., Billings C., Abdalla M., Korra A., Anderson D.E. (2023). In Vivo Assessment of High-Strength and Corrosion-Controlled Magnesium-Based Bone Implants. Bioengineering.

[15] Rahaman M.N., Day D.E., Bal B.S., Fu Q., Jung S.B., Bonewald L.F., Tomsia A.P. (2011). Bioactive Glass in Tissue Engineering. Acta Biomater..

[16] Jones J.R. (2013). Review of Bioactive Glass: From Hench to Hybrids. Acta Biomater..

[17] Chloros G.D., Prodromidis A.D., Wilson J., Giannoudis P.V. (2022). Fracture Fixation in Extremity Trauma with Carbon Fiber-Reinforced Polyetheretherketone (CFR-PEEK) Plates: Evidence Today. Eur. J. Trauma Emerg. Surg..

[18] Hamsho W., Raufi M.Y., Alnajjar M., Rhodes M., Hafeez U., Aurangzeb R. (2026). Carbon Fiber-Reinforced Polyetheretherketone (PEEK) vs. Titanium Plates for Upper Limb Fractures: A Systematic Review. Cureus.

[19] Willbold E., Weizbauer A., Loos A., Seitz J.-M., Angrisani N., Windhagen H., Reifenrath J. (2017). Magnesium Alloys: A Stony Pathway from Intensive Research to Clinical Reality. Different Test Methods and Approval-Related Considerations. J. Biomed. Mater. Res. A.

[20] Sukotjo C., Lima-Neto T.J., Santiago Júnior J.F., Faverani L.P., Miloro M. (2020). Is There a Role for Absorbable Metals in Surgery? A Systematic Review and Meta-Analysis of Mg/Mg Alloy Based Implants. Materials.

[21] Agarwal S., Curtin J., Duffy B., Jaiswal S. (2016). Biodegradable Magnesium Alloys for Orthopaedic Applications: A Review on Corrosion, Biocompatibility and Surface Modifications. Mater. Sci. Eng. C Mater. Biol. Appl..

[22] Zhao D., Witte F., Lu F., Wang J., Li J., Qin L. (2017). Current Status on Clinical Applications of Magnesium-Based Orthopaedic Implants: A Review from Clinical Translational Perspective. Biomaterials.

[23] Amukarimi S., Mozafari M. (2022). Biodegradable Magnesium Biomaterials-Road to the Clinic. Bioengineering.

[24] Thomas K.K., Zafar M.N., Pitt W.G., Husseini G.A. (2023). Biodegradable Magnesium Alloys for Biomedical Implants: Properties, Challenges, and Surface Modifications with a Focus on Orthopedic Fixation Repair. Appl. Sci..

[25] Martinez D.C., Dobkowska A., Marek R., Ćwieka H., Jaroszewicz J., Płociński T., Donik Č., Helmholz H., Luthringer-Feyerabend B., Zeller-Plumhoff B. (2023). In Vitro and in Vivo Degradation Behavior of Mg-0.45Zn-0.45Ca (ZX00) Screws for Orthopedic Applications. Bioact. Mater..

[26] Zhang C., Lin J., Nguyen N.-Y.T., Guo Y., Xu C., Seo C., Villafana E., Jimenez H., Chai Y., Guan R. (2020). Antimicrobial Bioresorbable Mg-Zn-Ca Alloy for Bone Repair in a Comparison Study with Mg-Zn-Sr Alloy and Pure Mg. ACS Biomater. Sci. Eng..

[27] Incesu A., Gungor A. (2020). Mechanical Properties and Biodegradability of Mg-Zn-Ca Alloys: Homogenization Heat Treatment and Hot Rolling. J. Mater. Sci. Mater. Med..

[28] Feng Y., Zhang Z., Jiang D., Khan M.A., Wang H., Zhao D., Wu Y., Wen L., Li J. (2026). A High-Performance Biodegradable Mg-Zn- Ca-Sn Alloy with Synergistic Improvement of Yield Strength and Corrosion Rate Achieved by Precise Control of Extrusion and Microstructural Design. Acta Biomater..

[29] Labmayr V., Suljevic O., Sommer N.G., Schwarze U.Y., Marek R.L., Brcic I., Foessl I., Leithner A., Seibert F.J., Herber V. (2024). Mg-Zn-Ca Alloy (ZX00) Screws Are Resorbed at a Mean of 2.5 Years after Medial Malleolar Fracture Fixation: Follow-up of a First-in-Humans Application and Insights from a Sheep Model. Clin. Orthop. Relat. Res..

[30] Atkinson H.D., Khan S., Lashgari Y., Ziegler A. (2019). Hallux Valgus Correction Utilising a Modified Short Scarf Osteotomy with a Magnesium Biodegradable or Titanium Compression Screws—A Comparative Study of Clinical Outcomes. BMC Musculoskelet. Disord..

[31] Weng W., Biesiekierski A., Li Y., Dargusch M., Wen C. (2021). A Review of the Physiological Impact of Rare Earth Elements and Their Uses in Biomedical Mg Alloys. Acta Biomater..

[32] Bian D., Chu X., Xiao J., Tong Z., Huang H., Jia Q., Liu J., Li W., Yu H., He Y. (2023). Design of Single-Phased Magnesium Alloys with Typically High Solubility Rare Earth Elements for Biomedical Applications: Concept and Proof. Bioact. Mater..

[33] Deng B., Dai Y., Lin J., Zhang D. (2022). Effect of Rolling Treatment on Microstructure, Mechanical Properties, and Corrosion Properties of WE43 Alloy. Materials.

[34] Dobatkin S., Martynenko N., Anisimova N., Kiselevskiy M., Prosvirnin D., Terentiev V., Yurchenko N., Salishchev G., Estrin Y. (2019). Mechanical Properties, Biodegradation, and Biocompatibility of Ultrafine Grained Magnesium Alloy WE43. Materials.

[35] Rendenbach C., Fischer H., Kopp A., Schmidt-Bleek K., Kreiker H., Stumpp S., Thiele M., Duda G., Hanken H., Beck-Broichsitter B. (2021). Improved in Vivo Osseointegration and Degradation Behavior of PEO Surface-Modified WE43 Magnesium Plates and Screws after 6 and 12 Months. Mater. Sci. Eng. C Mater. Biol. Appl..

[36] Torroni A., Xiang C., Witek L., Rodriguez E.D., Coelho P.G., Gupta N. (2017). Biocompatibility and Degradation Properties of WE43 Mg Alloys with and without Heat Treatment: In Vivo Evaluation and Comparison in a Cranial Bone Sheep Model. J. Craniomaxillofac. Surg..

[37] Czerniak C.W., Connon M.L., Wintersheimer E., Freitas E., MacRenaris K.W., Schaffer J.E., Griebel A.J., Zhou L., Sahoo D., Guillory R.J. (2026). Mg-Y-Nd Alloy Biocorrosion Behavior in Hyperlipidemia Models in Vitro and in Vivo. Acta Biomater..

[38] Yu Y., Lu H., Sun J. (2018). Long-Term in Vivo Evolution of High-Purity Mg Screw Degradation—Local and Systemic Effects of Mg Degradation Products. Acta Biomater..

[39] Han P., Cheng P., Zhang S., Zhao C., Ni J., Zhang Y., Zhong W., Hou P., Zhang X., Zheng Y. (2015). In Vitro and in Vivo Studies on the Degradation of High-Purity Mg (99.99 wt.%) Screw with Femoral Intracondylar Fractured Rabbit Model. Biomaterials.

[40] Huang S., Wang B., Zhang X., Lu F., Wang Z., Tian S., Li D., Yang J., Cao F., Cheng L. (2020). High-Purity Weight-Bearing Magnesium Screw: Translational Application in the Healing of Femoral Neck Fracture. Biomaterials.

[41] Zhang X., Li H. (2025). A Comprehensive Review on the Role of Lithium in Biodegradable Metals. Acta Biomater..

[42] Liu Y., Wu Y., Bian D., Gao S., Leeflang S., Guo H., Zheng Y., Zhou J. (2017). Study on the Mg-Li-Zn Ternary Alloy System with Improved Mechanical Properties, Good Degradation Performance and Different Responses to Cells. Acta Biomater..

[43] Wu J., Zhao D., Ohodnicki J.M., Lee B., Roy A., Yao R., Chen S., Dong Z., Heineman W.R., Kumta P.N. (2018). In Vitro and in Vivo Evaluation of Multiphase Ultrahigh Ductility Mg-Li-Zn Alloys for Cardiovascular Stent Application. ACS Biomater. Sci. Eng..

[44] Chen X.-B., Li C., Xu D. (2018). Biodegradation of Mg-14Li Alloy in Simulated Body Fluid: A Proof-of-Concept Study. Bioact. Mater..

[45] Tie D., Feyerabend F., Müller W.D., Schade R., Liefeith K., Kainer K.U., Willumeit R. (2013). Antibacterial Biodegradable Mg-Ag Alloys. Eur. Cells Mater..

[46] Yu K., Dai Y., Luo Z., Long H., Zeng M., Li Z., Zhu J., Cheng L., Zhang Y., Liu H. (2018). In Vitro and in Vivo Evaluation of Novel Biodegradable Mg-Ag-Y Alloys for Use as Resorbable Bone Fixation Implant. J. Biomed. Mater. Res. A.

[47] Ezechieli M., Ettinger M., König C., Weizbauer A., Helmecke P., Schavan R., Lucas A., Windhagen H., Becher C. (2016). Biomechanical Characteristics of Bioabsorbable Magnesium-Based (MgYREZr-Alloy) Interference Screws with Different Threads. Knee Surg. Sports Traumatol. Arthrosc..

[48] Siroros N., Merfort R., Liu Y., Praster M., Migliorini F., Maffulli N., Michalik R., Hildebrand F., Eschweiler J. (2023). Mechanical Properties of a Bioabsorbable Magnesium Interference Screw for Anterior Cruciate Ligament Reconstruction in Various Testing Bone Materials. Sci. Rep..

[49] Wang J., Wu Y., Li H., Liu Y., Bai X., Chau W., Zheng Y., Qin L. (2018). Magnesium Alloy Based Interference Screw Developed for ACL Reconstruction Attenuates Peri-Tunnel Bone Loss in Rabbits. Biomaterials.

[50] Castellani C., Lindtner R.A., Hausbrandt P., Tschegg E., Stanzl-Tschegg S.E., Zanoni G., Beck S., Weinberg A.-M. (2011). Bone-Implant Interface Strength and Osseointegration: Biodegradable Magnesium Alloy versus Standard Titanium Control. Acta Biomater..

[51] Ibrahim H., Esfahani S.N., Poorganji B., Dean D., Elahinia M. (2017). Resorbable Bone Fixation Alloys, Forming, and Post-Fabrication Treatments. Mater. Sci. Eng. C Mater. Biol. Appl..

[52] Kiani F., Wen C., Li Y. (2020). Prospects and Strategies for Magnesium Alloys as Biodegradable Implants from Crystalline to Bulk Metallic Glasses and Composites—A Review. Acta Biomater..

[53] Tian L., Sheng Y., Huang L., Chow D.H.-K., Chau W.H., Tang N., Ngai T., Wu C., Lu J., Qin L. (2018). An Innovative Mg/Ti Hybrid Fixation System Developed for Fracture Fixation and Healing Enhancement at Load-Bearing Skeletal Site. Biomaterials.

[54] Chen H., Wang Y., He L., Zhang X., Mei Y., Wu T., Wang J., Zheng Y., Tang H. (2025). Surface Engineering of Biodegradable Magnesium Alloys as Orthopedic Implant Materials: Recent Developments and Future Prospects. Coatings.

[55] Makurat-Kasprolewicz B., Matykina E. (2025). Frequency-Controlled AC-MAO Coatings with Ca, P, and Se on Magnesium: Toward Tailored Surfaces for Biodegradable Implants. Materials.

[56] Kopp A., Fischer H., Soares A.P., Schmidt-Bleek K., Leber C., Kreiker H., Duda G., Kröger N., van Gaalen K., Hanken H. (2023). Long-Term in Vivo Observations Show Biocompatibility and Performance of ZX00 Magnesium Screws Surface-Modified by Plasma-Electrolytic Oxidation in Göttingen Miniature Pigs. Acta Biomater..

[57] Wu G.-L., Yen C.-E., Hsu W.-C., Yeh M.-L. (2025). Incorporation of Cerium Oxide Nanoparticles into the Micro-Arc Oxidation Layer Promotes Bone Formation and Achieves Structural Integrity in Magnesium Orthopedic Implants. Acta Biomater..

[58] Tamay D.G., Gokyer S., Schmidt J., Vladescu A., Yilgor Huri P., Hasirci V., Hasirci N. (2022). Corrosion Resistance and Cytocompatibility of Magnesium-Calcium Alloys Modified with Zinc- or Gallium-Doped Calcium Phosphate Coatings. ACS Appl. Mater. Interfaces.

[59] Shi B., Li Y.R., Xu J., Zou J., Zhou Z., Jia Q., Jiang H.B., Liu K. (2024). Advances in Amelioration of Plasma Electrolytic Oxidation Coatings on Biodegradable Magnesium and Alloys. Heliyon.

[60] Chi J., Zhang H., Song S., Zhang W., He X., Nong Z., Cui X., Liu T., Man T. (2025). The Impact of Pre- and Post-Treatment Processes on Corrosion Resistance of Micro-Arc Oxidation Coatings on Mg Alloys: A Systematic Review. Materials.

[61] Cheng S., Wang W., Wang D., Li B., Zhou J., Zhang D., Liu L., Peng F., Liu X., Zhang Y. (2020). An in Vitro and in Vivo Comparison of Mg(OH)2-, MgF2- and HA-Coated Mg in Degradation and Osteointegration. Biomater. Sci..

[62] Kim S.-M., Jo J.-H., Lee S.-M., Kang M.-H., Kim H.-E., Estrin Y., Lee J.-H., Lee J.-W., Koh Y.-H. (2014). Hydroxyapatite-Coated Magnesium Implants with Improved in Vitro and in Vivo Biocorrosion, Biocompatibility, and Bone Response. J. Biomed. Mater. Res. A.

[63] Lan W., Li J., Lv Z., Liu S., Liang Z., Huang D., Wei X., Chen W. (2024). In Vitro Corrosion and Cytocompatibility of Mg-Zn-Ca Alloys Coated with FHA. Colloids Surf. B Biointerfaces.

[64] Gambaro S., Nascimento M.L., Shekargoftar M., Ravanbakhsh S., Sales V., Paternoster C., Bartosch M., Witte F., Mantovani D. (2022). Characterization of a Magnesium Fluoride Conversion Coating on Mg-2Y-1Mn-1Zn Screws for Biomedical Applications. Materials.

[65] Sun W., Zhang G., Tan L., Yang K., Ai H. (2016). The Fluoride Coated AZ31B Magnesium Alloy Improves Corrosion Resistance and Stimulates Bone Formation in Rabbit Model. Mater. Sci. Eng. C Mater. Biol. Appl..

[66] Witte F., Fischer J., Nellesen J., Vogt C., Vogt J., Donath T., Beckmann F. (2010). In Vivo Corrosion and Corrosion Protection of Magnesium Alloy LAE442. Acta Biomater..

[67] Makkar P., Kang H.J., Padalhin A.R., Park I., Moon B.-G., Lee B.T. (2018). Development and Properties of Duplex MgF2/PCL Coatings on Biodegradable Magnesium Alloy for Biomedical Applications. PLoS ONE.

[68] Saberi A., Bakhsheshi-Rad H.R., Abazari S., Ismail A.F., Sharif S., Ramakrishna S., Daroonparvar M., Berto F. (2021). A Comprehensive Review on Surface Modifications of Biodegradable Magnesium-Based Implant Alloy: Polymer Coatings Opportunities and Challenges. Coatings.

[69] Wong H.M., Yeung K.W.K., Lam K.O., Tam V., Chu P.K., Luk K.D.K., Cheung K.M.C. (2010). A Biodegradable Polymer-Based Coating to Control the Performance of Magnesium Alloy Orthopaedic Implants. Biomaterials.

[70] Jiang W., Tian Q., Vuong T., Shashaty M., Gopez C., Sanders T., Liu H. (2017). Comparison Study on Four Biodegradable Polymer Coatings for Controlling Magnesium Degradation and Human Endothelial Cell Adhesion and Spreading. ACS Biomater. Sci. Eng..

[71] Jiang Y., Wang B., Jia Z., Lu X., Fang L., Wang K., Ren F. (2017). Polydopamine Mediated Assembly of Hydroxyapatite Nanoparticles and Bone Morphogenetic Protein-2 on Magnesium Alloys for Enhanced Corrosion Resistance and Bone Regeneration. J. Biomed. Mater. Res. A.

[72] Dehghan-Chenar S., Zare H.R., Mohammadpour Z. (2024). Chitosan-ImH@γ-CD: A pH-Sensitive Smart Bio-Coating to Enhance the Corrosion Resistance of Magnesium Alloys in Bio-Implants. RSC Adv..

[73] Zeng R.-C., Cui L.-Y., Jiang K., Liu R., Zhao B.-D., Zheng Y.-F. (2016). In Vitro Corrosion and Cytocompatibility of a Microarc Oxidation Coating and poly(L-Lactic Acid) Composite Coating on Mg-1Li-1Ca Alloy for Orthopedic Implants. ACS Appl. Mater. Interfaces.

[74] Kannan M.B., Walter R., Yamamoto A., Khakbaz H., Blawert C. (2018). Electrochemical Surface Engineering of Magnesium Metal by Plasma Electrolytic Oxidation and Calcium Phosphate Deposition: Biocompatibility and in Vitro Degradation Studies. RSC Adv..

[75] Li Y., Zhao S., Li S., Ge Y., Wang R., Zheng L., Xu J., Sun M., Jiang Q., Zhang Y. (2019). Surface Engineering of Biodegradable Magnesium Alloys for Enhanced Orthopedic Implants. Small.

[76] Zhao Y., He P., Yao J., Li M., Wang B., Han L., Huang Z., Guo C., Bai J., Xue F. (2023). pH/NIR-Responsive and Self-Healing Coatings with Bacteria Killing, Osteogenesis, and Angiogenesis Performances on Magnesium Alloy. Biomaterials.

[77] Shang Z., Li D., Chen J., Wang M., Zhang B., Wang X., Ma B. (2021). The Role of Biodegradable Magnesium and Its Alloys in Anterior Cruciate Ligament Reconstruction: A Systematic Review and Meta-Analysis Based on Animal Studies. Front. Bioeng. Biotechnol..

[78] Saab M., Hildebrand F., Martel B., Blanchemain N. (2023). Osteoinductive Bone Morphogenic Protein, Collagen Scaffold, Calcium Phosphate Cement, and Magnesium-Based Fixation Enhance Anterior Cruciate Ligament Tendon Graft to Bone Healing in Animal Models: A Systematic Review. Arthroscopy.

[79] Cheng P., Han P., Zhao C., Zhang S., Zhang X., Chai Y. (2016). Magnesium Inference Screw Supports Early Graft Incorporation with Inhibition of Graft Degradation in Anterior Cruciate Ligament Reconstruction. Sci. Rep..

[80] Wang J., Xu J., Fu W., Cheng W., Chan K., Yung P.S.-H., Qin L. (2017). Biodegradable Magnesium Screws Accelerate Fibrous Tissue Mineralization at the Tendon-Bone Insertion in Anterior Cruciate Ligament Reconstruction Model of Rabbit. Sci. Rep..

[81] Wang J., Xu J., Song B., Chow D.H., Shu-Hang Yung P., Qin L. (2017). Magnesium (Mg) Based Interference Screws Developed for Promoting Tendon Graft Incorporation in Bone Tunnel in Rabbits. Acta Biomater..

[82] Xu C., Luo Y., Cai Z., Ji J., Guo Z., Cai Z., You C., Zhou Y., Chen Z., Zhang W. (2025). Magnesium Ions Attenuate Tendon Graft Fibrosis during Its Ligamentization after ACL Reconstruction through Modulation of Fibroblast to Myofibroblast Trans-Differentiation by Promoting PGE2 Secretion. Bioact. Mater..

[83] Farraro K.F., Sasaki N., Woo S.L.-Y., Kim K.E., Tei M.M., Speziali A., McMahon P.J. (2016). Magnesium Ring Device to Restore Function of a Transected Anterior Cruciate Ligament in the Goat Stifle Joint. J. Orthop. Res..

[84] Wang H., Kang H., Yao J., Cheng C.-K., Woo S.L.-Y. (2019). Evaluation of a Magnesium Ring Device for Mechanical Augmentation of a Ruptured ACL: Finite Element Analysis. Clin. Biomech..

[85] Hsu W.-C., Wu G.-L., Yeh M.-L. (2024). Fixation Technique of Biodegradable Magnesium Alloy Suture Anchor in the Rotator Cuff Repair of the Shoulder in a Goat Model: A Technical Note. BMC Musculoskelet. Disord..

[86] Chen B., Liang Y., Zhang J., Bai L., Xu M., Han Q., Han X., Xiu J., Li M., Zhou X. (2021). Synergistic Enhancement of Tendon-to-Bone Healing via Anti-Inflammatory and pro-Differentiation Effects Caused by Sustained Release of Mg2+/curcumin from Injectable Self-Healing Hydrogels. Theranostics.

[87] Wang T., Yu Z., Lin S., Chen Z., Jin H., Liang L., Zhang Z.-Y. (2024). 3D-Printed Mg-Incorporated PCL-Based Scaffolds Improves Rotator Cuff Tendon-Bone Healing through Regulating Macrophage Polarization. Front. Bioeng. Biotechnol..

[88] Sun M., Zhang W., Sun X., Yu K., He X., Zheng L., Ling Z., Duan K., Qi X., Liu Y. (2025). Magnesium Promoting OVX Rats’ Rotator Cuff Tear Repair with Relieving Stem Cell Senescence Effect. Exp. Cell Res..

[89] Zhang Z.-Z., Zhou Y.-F., Li W.-P., Jiang C., Chen Z., Luo H., Song B. (2019). Local Administration of Magnesium Promotes Meniscal Healing through Homing of Endogenous Stem Cells: A Proof-of-Concept Study. Am. J. Sports Med..

[90] Ranson W.A., Thurber L., Patel A.V., Schroen C., Cirino C.M., Denard P.J., Cagle P.J. (2025). Reconstruction of the Coracoclavicular Ligament Complex Utilizing an All-Suture Tape Cerclage Technique. Arthrosc. Tech..

[91] Wang J., Xu J., Wang X., Sheng L., Zheng L., Song B., Wu G., Zhang R., Yao H., Zheng N. (2021). Magnesium-Pretreated Periosteum for Promoting Bone-Tendon Healing after Anterior Cruciate Ligament Reconstruction. Biomaterials.

[92] Delsmann M.M., Stürznickel J., Kertai M., Stücker R., Rolvien T., Rupprecht M. (2023). Radiolucent Zones of Biodegradable Magnesium-Based Screws in Children and Adolescents-a Radiographic Analysis. Arch. Orthop. Trauma Surg..

[93] Sun Y., Wu H., Wang W., Zan R., Peng H., Zhang S., Zhang X. (2019). Translational Status of Biomedical Mg Devices in China. Bioact. Mater..

[94] Windhagen H., Radtke K., Weizbauer A., Diekmann J., Noll Y., Kreimeyer U., Schavan R., Stukenborg-Colsman C., Waizy H. (2013). Biodegradable Magnesium-Based Screw Clinically Equivalent to Titanium Screw in Hallux Valgus Surgery: Short Term Results of the First Prospective, Randomized, Controlled Clinical Pilot Study. Biomed. Eng. Online.

[95] Plaass C., von Falck C., Ettinger S., Sonnow L., Calderone F., Weizbauer A., Reifenrath J., Claassen L., Waizy H., Daniilidis K. (2018). Bioabsorbable Magnesium versus Standard Titanium Compression Screws for Fixation of Distal Metatarsal Osteotomies—3 Year Results of a Randomized Clinical Trial. J. Orthop. Sci..

[96] Gazit T., Robinson D., Khawalde K., Eisa M., Qassem K., Heller E., Yassin M. (2024). Foot Surgery Using Resorbable Magnesium Screws. J. Foot Ankle Surg..

[97] Kose O., Turan A., Unal M., Acar B., Guler F. (2018). Fixation of Medial Malleolar Fractures with Magnesium Bioabsorbable Headless Compression Screws: Short-Term Clinical and Radiological Outcomes in Eleven Patients. Arch. Orthop. Trauma Surg..

[98] Könneker S., Schächinger U., Vogt P.M. (2023). Magnesium-Based Compression Screws in Acute Scaphoid Fractures and Nonunions. World J. Surg..

[99] Zhao D., Huang S., Lu F., Wang B., Yang L., Qin L., Yang K., Li Y., Li W., Wang W. (2016). Vascularized Bone Grafting Fixed by Biodegradable Magnesium Screw for Treating Osteonecrosis of the Femoral Head. Biomaterials.

[100] Yu X., Zhao D., Huang S., Wang B., Zhang X., Wang W., Wei X. (2015). Biodegradable Magnesium Screws and Vascularized Iliac Grafting for Displaced Femoral Neck Fracture in Young Adults. BMC Musculoskelet. Disord..

[101] Stürznickel J., Delsmann M.M., Jungesblut O.D., Stücker R., Knorr C., Rolvien T., Kertai M., Rupprecht M. (2021). Safety and Performance of Biodegradable Magnesium-Based Implants in Children and Adolescents. Injury.

[102] Gigante A., Setaro N., Rotini M., Finzi S.S., Marinelli M. (2018). Intercondylar Eminence Fracture Treated by Resorbable Magnesium Screws Osteosynthesis: A Case Series. Injury.

[103] Ernstberger T., Buchhorn G., Heidrich G. (2009). Artifacts in Spine Magnetic Resonance Imaging due to Different Intervertebral Test Spacers: An in Vitro Evaluation of Magnesium versus Titanium and Carbon-Fiber-Reinforced Polymers as Biomaterials. Neuroradiology.

[104] Waelti S.L., Wildermuth S., Willems E.P., Fischer T., Dietrich T.J., Leschka S., Matissek C., Krebs T., Markart S. (2023). Prospective Evaluation of Magnetic Resonance Imaging Features of Magnesium-Based Alloy Screw Resorption in Pediatric Fractures. J. Clin. Med..

[105] Espiritu J., Berangi M., Cwieka H., Iskhakova K., Kuehne A., Florian Wieland D.C., Zeller-Plumhoff B., Niendorf T., Willumeit-Römer R., Seitz J.-M. (2023). Radiofrequency Induced Heating of Biodegradable Orthopaedic Screw Implants during Magnetic Resonance Imaging. Bioact. Mater..

[106] Berangi M., Waiczies H., Saha N., Shalikar S., Majd M.S., Niendorf T. (2026). Degradation of Biodegradable Orthopedic Screws Does (not) Elevate Tissue Heating under Magnetic Resonance Imaging: Implications for next Generation Implants. Bioact. Mater..

[107] Waelti S.L., Markart S., Willems E.P., Fischer T., Dietrich T.J., Ditchfield M., Matissek C., Krebs T. (2022). Radiographic Features of Magnesium-Based Bioabsorbable Screw Resorption in Paediatric Fractures. Pediatr. Radiol..

[108] Kuhlmann J., Bartsch I., Willbold E., Schuchardt S., Holz O., Hort N., Höche D., Heineman W.R., Witte F. (2013). Fast Escape of Hydrogen from Gas Cavities around Corroding Magnesium Implants. Acta Biomater..

[109] Schroen C.A., Nasser P., Laudier D., Boecker A.H., Cagle P.J., Hausman M.R. (2026). Second Harmonic Generation Imaging as a Virtual Biopsy for Upper Extremity Nerve Injuries: A Cadaver Study. Hand.

[110] Schroen C.A., Nasser P., Boecker A.H., Cagle P.J., Hausman M.R. (2026). Second Harmonic Generation Imaging Reveals Extent of Damage in Acute Nerve Stretch Injuries in Rats. J. Surg. Res..

[111] Gluck M.J., Beck C.M., Skodras A., Bernstein Z.L., Rubin T.A., Hausman M.R., Cagle P.J. (2023). Second Harmonic Generation Microscopy as a Novel Intraoperative Assessment of Rat Median Nerve Injury. J. Hand Surg. Am..

[112] Sivaguru M., Durgam S., Ambekar R., Luedtke D., Fried G., Stewart A., Toussaint K.C. (2010). Quantitative Analysis of Collagen Fiber Organization in Injured Tendons Using Fourier Transform-Second Harmonic Generation Imaging. Opt. Express.

[113] Qin Y., Wen P., Guo H., Xia D., Zheng Y., Jauer L., Poprawe R., Voshage M., Schleifenbaum J.H. (2019). Additive Manufacturing of Biodegradable Metals: Current Research Status and Future Perspectives. Acta Biomater..

[114] Kopp A., Derra T., Müther M., Jauer L., Schleifenbaum J.H., Voshage M., Jung O., Smeets R., Kröger N. (2019). Influence of Design and Postprocessing Parameters on the Degradation Behavior and Mechanical Properties of Additively Manufactured Magnesium Scaffolds. Acta Biomater..

[115] Liu J., Liu B., Min S., Yin B., Peng B., Yu Z., Wang C., Ma X., Wen P., Tian Y. (2022). Biodegradable Magnesium Alloy WE43 Porous Scaffolds Fabricated by Laser Powder Bed Fusion for Orthopedic Applications: Process Optimization, in Vitro and in Vivo Investigation. Bioact. Mater..

[116] Dong J., Lin P., Putra N.E., Tümer N., Leeflang M.A., Huan Z., Fratila-Apachitei L.E., Chang J., Zadpoor A.A., Zhou J. (2022). Extrusion-Based Additive Manufacturing of Mg-Zn/bioceramic Composite Scaffolds. Acta Biomater..

[117] Dong J., Li Y., Lin P., Leeflang M.A., van Asperen S., Yu K., Tümer N., Norder B., Zadpoor A.A., Zhou J. (2020). Solvent-Cast 3D Printing of Magnesium Scaffolds. Acta Biomater..

[118] Zhou J., Georgas E., Su Y., Zhou J., Kröger N., Benn F., Kopp A., Qin Y.-X., Zhu D. (2023). Evolution from Bioinert to Bioresorbable: In Vivo Comparative Study of Additively Manufactured Metal Bone Scaffolds. Adv. Sci..

[119] Witte F., Fischer J., Nellesen J., Crostack H.-A., Kaese V., Pisch A., Beckmann F., Windhagen H. (2006). In Vitro and in Vivo Corrosion Measurements of Magnesium Alloys. Biomaterials.

[120] Walker J., Shadanbaz S., Kirkland N.T., Stace E., Woodfield T., Staiger M.P., Dias G.J. (2012). Magnesium Alloys: Predicting in Vivo Corrosion with in Vitro Immersion Testing. J. Biomed. Mater. Res. B Appl. Biomater..

[121] Witecka A., Bogucka A., Yamamoto A., Máthis K., Krajňák T., Jaroszewicz J., Święszkowski W. (2016). In Vitro Degradation of ZM21 Magnesium Alloy in Simulated Body Fluids. Mater. Sci. Eng. C Mater. Biol. Appl..

[122] (2018). Biological Evaluation of Medical Devices—Part 1: Evaluation and Testing Within a Risk Management Process.

[123] Scheideler L., Füger C., Schille C., Rupp F., Wendel H.-P., Hort N., Reichel H.P., Geis-Gerstorfer J. (2013). Comparison of Different in Vitro Tests for Biocompatibility Screening of Mg Alloys. Acta Biomater..

[124] Könneker S., Krockenberger K., Pieh C., von Falck C., Brandewiede B., Vogt P.M., Kirschner M.H., Ziegler A. (2019). Comparison of SCAphoid Fracture Osteosynthesis by MAGnesium-Based Headless Herbert Screws with Titanium Herbert Screws: Protocol for the Randomized Controlled SCAMAG Clinical Trial. BMC Musculoskelet. Disord..

[125] Zhang H., Zhang P., Shen X., Han J., Wang H., Qin H., Wang B., Qian J., Udduttula A., Luo R. (2025). A Cross-Linked Coating Loaded with Antimicrobial Peptides for Corrosion Control, Early Antibacterial, and Sequential Osteogenic Promotion on a Magnesium Alloy as Orthopedic Implants. Acta Biomater..

[126] Zhang L., Pei J., Wang H., Shi Y., Niu J., Yuan F., Huang H., Zhang H., Yuan G. (2017). Facile Preparation of Poly(lactic Acid)/brushite Bilayer Coating on Biodegradable Magnesium Alloys with Multiple Functionalities for Orthopedic Application. ACS Appl. Mater. Interfaces.

[127] Gharehdaghi N., Nokhbatolfoghahaei H., Khojasteh A. (2024). 4D Printing of Smart Scaffolds for Bone Regeneration: A Systematic Review. Biomed. Mater..

[128] Wan Z., Zhang P., Liu Y., Lv L., Zhou Y. (2020). Four-Dimensional Bioprinting: Current Developments and Applications in Bone Tissue Engineering. Acta Biomater..

[129] Chen X., Han S., Wu W., Wu Z., Yuan Y., Wu J., Liu C. (2022). Harnessing 4D Printing Bioscaffolds for Advanced Orthopedics. Small.

[130] Zhang J., Sun N., Liu W., Lu S., Jiang L., Zhang J., Zhang B., Lin Z., Wang T., Li L. (2026). MAO/SA Composite Coating on Magnesium Alloy: Corrosion Resistance Improvement and Time-Programmed Ca2+/Mg2+ Release for Bone Repair. Colloids Surf. B Biointerfaces.

[131] Schroen C.A., Siniakowicz C., Nasser P., Hausman M.R., Cagle P.J. (2026). Earlier Functional Recovery with an Amniochorionic Membrane Allograft Nerve Wrap after in-Continuity Stretch Injury in Rat Median Nerves. J. Plast. Reconstr. Aesthet. Surg..

[132] McClendon D.C., Su J., Smith D.W. (2023). Human Amniotic Allograft in Hand Surgery. J. Hand Surg. Am..

[133] Song H.S., Gong H.-G., Lee H.-J., Kim H., Tae K.-S. (2025). The Optimal Fibular Strut Bone Graft Fixation Angle for Unstable Proximal Humerus Fractures: A Finite Element Analysis. Bioengineering.

[134] Asoglu M.M., Kizilkaya V., Levent A., Celik H.K., Kose O., Rennie A.E.W. (2026). Medial Malleolar Fracture Fixation with Stainless Steel, Titanium, Magnesium, and PLGA Screws: A Finite Element Analysis. J. Funct. Biomater..

[135] Cho S.-M., Yang B.-E., Kim W.-H., Park S.-Y., On S.-W., Lee J.-H., Byun S.-H. (2024). Biomechanical Stability of Magnesium Plate and Screw Fixation Systems in LeFort I Osteotomy: A Three-Dimensional Finite Element Analysis. Maxillofac. Plast. Reconstr. Surg..

[136] Gastaldi D., Sassi V., Petrini L., Vedani M., Trasatti S., Migliavacca F. (2011). Continuum Damage Model for Bioresorbable Magnesium Alloy Devices—Application to Coronary Stents. J. Mech. Behav. Biomed. Mater..

[137] Debusschere N., Segers P., Dubruel P., Verhegghe B., De Beule M. (2016). A Computational Framework to Model Degradation of Biocorrodible Metal Stents Using an Implicit Finite Element Solver. Ann. Biomed. Eng..

[138] Boland E.L., Shine C.J., Kelly N., Sweeney C.A., McHugh P.E. (2016). A Review of Material Degradation Modelling for the Analysis and Design of Bioabsorbable Stents. Ann. Biomed. Eng..

[139] Amerinatanzi A., Mehrabi R., Ibrahim H., Dehghan A., Shayesteh Moghaddam N., Elahinia M. (2018). Predicting the Biodegradation of Magnesium Alloy Implants: Modeling, Parameter Identification, and Validation. Bioengineering.

[140] Luo Y., Liu F., Chen Z., Luo Y., Li W., Wang J. (2024). A Magnesium Screw with Optimized Geometry Exhibits Improved Corrosion Resistance and Favors Bone Fracture Healing. Acta Biomater..

[141] Pohl N., Priebe D., AlBaraghtheh T., Schimek S., Wieland D.C.F., Krüger D., Trostorff S., Willumeit-Römer R., Köhl R., Zeller-Plumhoff B. (2025). Computational Modelling of Bone Growth and Mineralization Surrounding Biodegradable Mg-Based and Permanent Ti Implants. Comput. Struct. Biotechnol. J..

[142] Manescu Paltanea V., Antoniac I., Antoniac A., Laptoiu D., Paltanea G., Ciocoiu R., Nemoianu I.V., Gruionu L.G., Dura H. (2023). Bone Regeneration Induced by Patient-Adapted Mg Alloy-Based Scaffolds for Bone Defects: Present and Future Perspectives. Biomimetics.

[143] Salehi M., Neo D.W.K., Rudel V., Stautner M., Ganser P., Zhang S.X., Seet H.L., Nai M.L.S. (2024). Digital Manufacturing of Personalized Magnesium Implants Through Binder Jet Additive Manufacturing and Automated Post Machining. J. Magnes. Alloys.

[144] Lv Z., Peng B., Ye Y., Xu H., Cai X., Liu J., Dai J., Bian Y., Wen P., Weng X. (2025). Bolstered Bone Regeneration by Multiscale Customized Magnesium Scaffolds with Hierarchical Structures and Tempered Degradation. Bioact. Mater..

[145] Wang Y., Fu P., Wang N., Peng L., Kang B., Zeng H., Yuan G., Ding W. (2020). Challenges and Solutions for the Additive Manufacturing of Biodegradable Magnesium Implants. Engineering.

[146] Hermawan H. (2018). Updates on the Research and Development of Absorbable Metals for Biomedical Applications. Prog. Biomater..

[147] AlOmran A.K., Alosaimi N., Alshaikhi A.A., Bakhurji O.M., Alzahrani K.J., Salloot B.Z., Alabduladhem T.O., AlMulhim A.I., Alumran A. (2024). Burden of Routine Orthopedic Implant Removal: A Single Center Retrospective Study. World J. Orthop..

[148] Sun Q., Zhou Y., Zhang A., Wu J., Tan L., Guo S. (2024). The Immunomodulatory Effects and Mechanisms of Magnesium-Containing Implants in Bone Regeneration: A Review. J. Magnes. Alloys.

[149] Ma Y., Chen H., Yuan Y., Yang G., Li J. (2026). Bioactive Components of Degradation Products from Biomedical Magnesium Alloys: Interactions with the In Vivo Microenvironment. Coatings.

[150] Chen L., Zhu J., Ge N., Liu Y., Yan Z., Liu G., Li Y., Wu G., Wang Y., Qiu T. (2025). A Biodegradable Magnesium Alloy Promotes Subperiosteal Osteogenesis via Interleukin-10-Dependent Macrophage Immunomodulation. Biomaterials.

[151] Ben Amara H., Martinez D.C., Iskhakova K., Emanuelsson L., Norlindh B. (2025). Multifaceted Bone Response to Immunomodulatory Magnesium Implants: Osteopromotion at the Interface and Adipogenesis in the Bone Marrow. Biomaterials.

[152] Koehne N.H., Locke A.R., Schroen C.A., Kator J., Awah C.E., Hausman M.R. (2026). Randomized Controlled Trials Evaluating Treatments for Carpometacarpal Arthritis Are Statistically Fragile: A Systematic Review. Hand.

[153] Forrester L.A., McCormick K.L., Bonsignore-Opp L., Tedesco L.J., Baranek E.S., Jang E.S., Tyler W.K. (2021). Statistical Fragility of Surgical Clinical Trials in Orthopaedic Trauma. J. Am. Acad. Orthop. Surg. Glob. Res. Rev..

[154] Tiao J., Song J., Hoang R., Restrepo Mejia M., Rajjoub R., Bhadouria N., Koehne N.H., Locke A.R., Yendluri A., Huang J.J. (2026). The Statistical Fragility of Lumbar Disc Arthroplasty vs Lumbar Fusion: A Systematic Review of Randomized Controlled Trials. Glob. Spine J..

[155] Checketts J.X., Scott J.T., Meyer C., Horn J., Jones J., Vassar M. (2018). The Robustness of Trials That Guide Evidence-Based Orthopaedic Surgery. J. Bone Jt. Surg. Am..

[156] Lauck B.J., Reynolds A.W., van der List J.P., Deivert K., Dean R.S., Trasolini N.A., Waterman B.R. (2025). Bioactive and Bioinductive Implants Are Increasingly Used in Orthopaedic Sports Medicine but Adequately Controlled Studies Are Needed: A Scoping Review. Arthroscopy.

[157] Poursalehian M., Sahebi M., Tajvidi M., Sabaghian A., Asgari A.-M., Tabaie S.A., Bhandari M., Hoveidaei A.H. (2025). Beyond the Usual Significance: Fragility Indices of Randomized Controlled Trials in Top General Orthopaedic Journals. J. Am. Acad. Orthop. Surg..

[158] Luo Y., Zhang C., Wang J., Liu F., Chau K.W., Qin L., Wang J. (2021). Clinical Translation and Challenges of Biodegradable Magnesium-Based Interference Screws in ACL Reconstruction. Bioact. Mater..

[159] Thormann U., Alt V., Heimann L., Gasquere C., Heiss C., Szalay G., Franke J., Schnettler R., Lips K.S. (2015). The Biocompatibility of Degradable Magnesium Interference Screws: An Experimental Study with Sheep. BioMed Res. Int..

